# The Cell Wall Polymer Lipoteichoic Acid Becomes Nonessential in *Staphylococcus aureus* Cells Lacking the ClpX Chaperone

**DOI:** 10.1128/mBio.01228-16

**Published:** 2016-08-09

**Authors:** Kristoffer T. Bæk, Lisa Bowman, Charlotte Millership, Mia Dupont Søgaard, Volkhard Kaever, Pia Siljamäki, Kirsi Savijoki, Pekka Varmanen, Tuula A. Nyman, Angelika Gründling, Dorte Frees

**Affiliations:** a Department of Veterinary Disease Biology, Faculty of Health and Medical Sciences, University of Copenhagen, Frederiksberg, Denmark; b Section of Microbiology and MRC Centre for Molecular Bacteriology and Infection, Imperial College London, London, United Kingdom; c Research Core Unit Metabolomics, Hannover Medical School, Hannover, Germany; d Department of Food and Environmental Sciences, University of Helsinki, Helsinki, Finland; e Institute of Biotechnology, Proteomics Unit, University of Helsinki, Helsinki, Finland

## Abstract

Lipoteichoic acid (LTA) is an important cell wall component of Gram-positive bacteria and a promising target for the development of vaccines and antimicrobial compounds against *Staphylococcus aureus*. Here we demonstrate that mutations in the conditionally essential *ltaS* (LTA synthase) gene arise spontaneously in an *S. aureus* mutant lacking the ClpX chaperone. A wide variety of *ltaS* mutations were selected, and among these, a substantial portion resulted in premature stop codons and other changes predicted to abolish LtaS synthesis. Consistent with this assumption, the *clpX ltaS* double mutants did not produce LTA, and genetic analyses confirmed that LTA becomes nonessential in the absence of the ClpX chaperone. In fact, inactivation of *ltaS* alleviated the severe growth defect conferred by the *clpX* deletion. Microscopic analyses showed that the absence of ClpX partly alleviates the septum placement defects of an LTA-depleted strain, while other phenotypes typical of LTA-negative *S. aureus* mutants, including increased cell size and decreased autolytic activity, are retained. In conclusion, our results indicate that LTA has an essential role in septum placement that can be bypassed by inactivating the ClpX chaperone.

## IMPORTANCE

Lipoteichoic acid is an essential component of the *Staphylococcus aureus* cell envelope and an attractive target for the development of vaccines and antimicrobials directed against antibiotic-resistant Gram-positive bacteria such as methicillin-resistant *S. aureus* and vancomycin-resistant enterococci. In this study, we showed that the lipoteichoic acid polymer is essential for growth of *S. aureus* only as long as the ClpX chaperone is present in the cell. Our results indicate that lipoteichoic acid and ClpX play opposite roles in a pathway that controls two key cell division processes in *S. aureus*, namely, septum formation and autolytic activity. The discovery of a novel functional connection in the genetic network that controls cell division in *S. aureus* may expand the repertoire of possible strategies to identify compounds or compound combinations that kill antibiotic-resistant *S. aureus*.

## INTRODUCTION

*Staphylococcus aureus* remains a leading cause of hospital- and community-acquired skin and soft tissue infections as well as of life-threatening invasive diseases (1). While β-lactams such as penicillin and methicillin were once effective antibiotics in treating staphylococcal infections, the spread of methicillin-resistant *S. aureus* (MRSA) has made treatment increasingly difficult (2). The profound ability of *S. aureus* to acquire resistance to clinically relevant antibiotics has prompted interest in identifying targets that can be used for the development of novel antibiotics with activity against MRSA (3).

The cell wall polymer lipoteichoic acid (LTA) has been proposed as an attractive target for the development of both vaccines and antimicrobial compounds directed against *S. aureus* (reviewed in reference 4). LTA is found abundantly in the cell wall of many Gram-positive bacteria and is defined as an alditol phosphate-containing polymer that is attached to the outer side of the cytoplasmic membrane via a lipid anchor (4). Gram-positive bacteria synthesize different types of LTA that, based on their chemical structures, are grouped into different types (4, 5). *S. aureus*, like many other species of *Firmicutes*, produces type I LTA consisting of an unbranched 1-3-linked glycerolphosphate (GroP) backbone chain. The addition of GroP residues to the tip of the growing LTA chain takes place at the outer surface of bacterial membranes and is catalyzed by the LTA synthase (LtaS) enzyme (6). The exact cellular function of LTA remains unknown, but, importantly, LTA is essential for growth of *S. aureus* under standard laboratory conditions (6, 7). Depletion of LtaS results in cell enlargement, aberrant positioning of division septa, and, ultimately, cell lysis, pointing to a functional link between LTA and the cell division process (6, 8). Consistent with this idea, LtaS of *S. aureus* was recently shown to interact with numerous early and late cell division proteins as well as with enzymes involved in peptidoglycan synthesis (9). In the rod-shaped *Firmicutes* species *Bacillus subtilis* and *Listeria monocytogenes*, inactivation of LTA synthesis also interferes with cell division, which is evidenced by cell filamentation and delocalization of the FtsZ cell division initiator protein (10, 11). A functional link between LTA synthesis and cell division therefore seems to be conserved within the family of *Firmicutes*. Other observations, however, have indicated that the essential role of LTA in *S. aureus* is related, at least partly, to osmoprotection. First, LTA-deficient *S. aureus* strains can be propagated under osmotically stabilizing conditions (8). Second, growth of an *S. aureus* ltaS mutant was rescued by inactivation of GdpP, a phosphodiesterase that hydrolyzes the essential signaling molecule cyclic-di-AMP, which is implicated in the control of potassium transport, an important ion in osmotic homeostasis (7, 8). In fact, type I LTA was recently proposed to share functionality with the osmoregulated periplasmic glucans (OPG) that accumulate in the periplasm of Gram-negative bacteria under low-osmolarity conditions (4).

The highly conserved ClpX chaperone has a dual role in the cell, as it both targets proteins for degradation by associating with the ClpP peptidase and, independently of ClpP, facilitates protein folding and interactions (12). In *S. aureus*, inactivation of *clpX* severely reduced virulence in both localized and systemic models of infections, suggesting that ClpX is indispensable for *S. aureus* pathogenesis (13, 14). Consistent with this observation, ClpX controls, by an unknown mechanism, the transcription of a number of major virulence genes and the translation of protein A and the global virulence regulator Rot (14–16). ClpX belongs to the Hsp100 family of heat shock proteins that become critical under conditions of heat stress and other stresses conferring protein-folding stress (12). Surprisingly, inactivation of *clpX* in *S. aureus* improved survival at high temperatures, indicating that ClpX does not function as a classical heat shock chaperone in this bacterium (14). Consistent with this notion, *clpX* transcription is not inducible by heat stress in *S. aureus* (14). Inactivation of ClpX in *S. aureus* did, however, severely impair growth at 30°C and below (14). The molecular mechanism underlying this phenotype is unknown and is not shared by a mutant lacking ClpP, suggesting that it is the ClpP-independent chaperone activity of ClpX that is important for growth of *S. aureus* at 30°C (14). In the present study, we used a whole-genome sequencing approach to characterize compensatory mutations that arise spontaneously with high frequency when the *clpX* deletion mutant is propagated at 30°C and below. Intriguingly, the most frequently detected type of mutation was the loss-of-function mutation in the *ltaS* gene. Essentiality of LtaS in the isogenic wild-type strains was confirmed, thus demonstrating that *S. aureus* can grow without LTA in the absence of the ClpX chaperone. To gain insight into the mechanisms underlying the requirement for LTA, a phenotypic characterization of the *clpX ltaS* double mutant was performed. From this analysis, we conclude that disruption of *clpX* compensates for the absence of LTA by a c-di-AMP-independent pathway that appears to be linked to cell division.

## RESULTS

### Inactivation of ClpX confers a severe growth defect at 30°C that is independent of the strain background and of ClpP.

**FIG 1:**
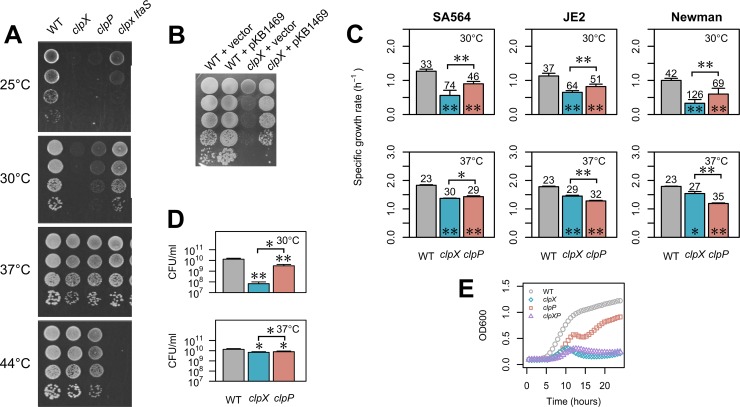
ClpX is required for growth at low temperatures in *S. aureus*. (A) ClpX is required for growth at low but not high temperatures. Wild-type (WT) *S. aureus* strain SA564 and the indicated derivatives were grown in TSB at 37°C. At an OD_600_ of 0.5, the cultures were diluted 10^1^-, 10^2^-, 10^3^-, and 10^4^-fold, and 5 μl of each dilution was spotted on TSA plates that were subsequently incubated at the indicated temperatures. (B) The *clpX*-related growth defect can be complemented. Wild-type *S. aureus* strain SA564 and the corresponding *clpX* mutant harboring either a plasmid expressing ClpX under the control of an anhydrotetracycline (AHT)-inducible promoter (pKB1469) or empty vector were grown in TSB at 37°C and diluted 10^1^-, 10^2^-, 10^3^-, 10^4^-, and 10^5^-fold. A 5-μl volume of each dilution was spotted on TSA plates containing 25 ng/ml AHT that were subsequently incubated at 30°C. (C) Growth rates of wild-type and mutant strains at 30°C and 37°C. Cultures of SA564, JE2, or Newman strains and the corresponding *clpX* and *clpP* mutants were grown overnight at 37°C and diluted into 300 μl TSB, and growth was measured at 30°C in a Bioscreen C instrument. Values represent mean specific growth rates (per hour) ± standard deviations determined in at least three biological replicates. Numbers above each bar indicate mean doubling times in minutes. Statistically significant differences between each mutant and the corresponding wild type or between pairs of mutants (as indicated by the horizontal bar) are indicated with asterisks (*, *P* < 0.05; **, *P* < 0.001). (D) Final yield (CFU per milliliter) at 30°C and 37°C. Overnight cultures of SA564 and the indicated mutant strains were diluted 1:200 in TSB and grown at the indicated temperature for 24 h. Cultures were diluted, plated, and incubated at 37°C. Values represent mean CFU per milliliter ± standard deviations from at least three biological replicates. Statistically significant differences between each mutant and the wild type or between the two mutants (as indicated by the horizontal bar) are indicated with asterisks (*, *P* < 0.05; **, *P* < 0.001). (E) Growth curve of *clpX clpP* double mutant. *S. aureus* strain 8325-4 and corresponding *clpX*, *clpP*, and *clpX clpP* strains were grown overnight at 37°C and diluted into 300 μl TSB, and growth was measured at 30°C in a Bioscreen C instrument. Values represent mean OD readings ± standard deviations (error bars are smaller than the symbols) of the results from three biological replicates.

We previously reported that an *S. aureus* clpX mutant forms colonies of much reduced size at 30°C (14). The original *clpX* mutant was constructed in laboratory *S. aureus* strain 8325-4, which has very low activity with respect to the SigB stress response regulator (14, 17). We therefore first set out to assess if the cold-sensitive phenotype of the *clpX* mutant is conserved in SigB-proficient *S. aureus* strains. The effect on growth of a *clpX* deletion was examined in more detail in four different backgrounds, namely, widely used laboratory strains 8325-4 and Newman, low-passage-number clinical isolate SA564, and JE2, an erythromycin-sensitive derivative of the USA300 LAC community-acquired MRSA strain. In all strain backgrounds, the *clpX* deletion mutants formed colonies that were slightly smaller than wild-type colonies at a temperature of 37°C or higher (Fig. 1A and data not shown). When the temperature was reduced to 30°C or 25°C, the *clpX* mutants formed barely visible colonies, demonstrating that deletion of *clpX* confers a growth defect to *S. aureus* that increases with decreasing temperatures in all strain backgrounds (Fig. 1A and data not shown). Introduction of a *clpX*-complementing plasmid enables the *clpX* mutant strain to form colonies of wild-type size (Fig. 1B). Growth assays in liquid medium at 30°C showed that the *clpX* mutants exhibited 42% to 67% lower exponential growth rates (depending on strain background; Fig. 1C) and that the *clpX* mutant reached a much lower final density (measured as CFU per milliliter or optical density [OD]; Fig. 1D and E) independently of the size and growth status of the inoculum (see Fig. S1 in the supplemental material). At 37°C, the growth defect of the *clpX* mutants was much less severe, as the cultures reached roughly the same final CFU count per milliliter as the respective wild-type strains and the exponential growth rates were reduced only slightly (14% to 25%; Fig. 1C and D).

ClpX can associate with ClpP to form the ClpXP protease. To investigate if ClpX contributes to growth at low temperatures via a ClpP-dependent pathway, we examined the effect of a *clpP* deletion on growth. We found that although the *clpP* mutants grew slower than the parental strains, similar reductions in exponential growth rates were observed at 30°C (27% to 29%) and 37°C (22% to 33%; Fig. 1C). Additionally, the temperature had little effect on the endpoint CFU count per milliliter (Fig. 1D). Thus, the growth defect conferred by the *clpP* deletion does not mimic the temperature-dependent effect of the *clpX* deletion, suggesting that it is the ClpP-independent chaperone activity which is required for growth at temperatures below the optimum. The ClpC chaperone is an alternative substrate recognition factor for ClpP, and, to rule out the possibility that the impaired growth of the *clpX* mutant was caused by an abnormal activity of ClpCP (due to the absence of ClpX), we constructed an *S. aureus* 8325-4 *clpX clpP* double mutant (KB1506) and compared its growth with that of the 8325-4 *clpX* single mutant. As seen in Fig. 1E, the *clpX clpP* double mutant exhibited a growth defect similar to that of the *clpX* mutant, supporting the notion that the *clpX* growth phenotype is not mediated via ClpCP. In summary, inactivation of the ClpX chaperone activity in *S. aureus* confers a cold-sensitive phenotype to *S. aureus* that is independent of ClpP and of the strain background.

### Loss-of-function mutations in the *ltaS* gene arise spontaneously in *clpX* mutants grown at suboptimal temperatures.

**FIG 2:**
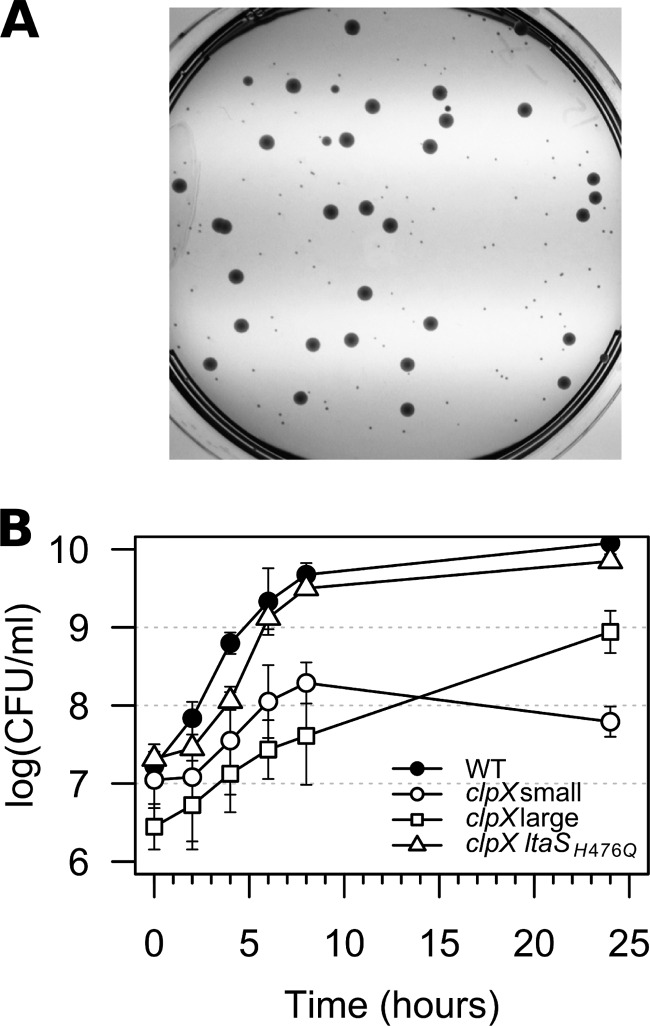
Spontaneous suppressor mutants restore growth of the *S. aureus* clpX mutant. (A) Faster-growing suppressor mutants appeared spontaneously in *clpX* cultures grown at low temperature. An overnight culture of the *S. aureus* SA564 *clpX* mutant was grown at 30°C in TSB, diluted and spread on a TSA plate, and incubated at 30°C for 48 h. (B) Growth of the *S. aureus* SA564 wild-type strain, the SA564 *clpX* strain, and the 564-25-2 (*ltaS_H476Q_*) spontaneous suppressor strain at 30°C in TSB measured as CFU per milliliter. Colony formation was assessed by incubating plates for 48 h at 30°C. “*clpX* small” indicates the count of small, normal-sized colonies, and “*clpX* large” indicates the count of large suppressor colonies. Values represent means of the results from three replicates, and error bars indicate standard deviations.

**FIG 3:**
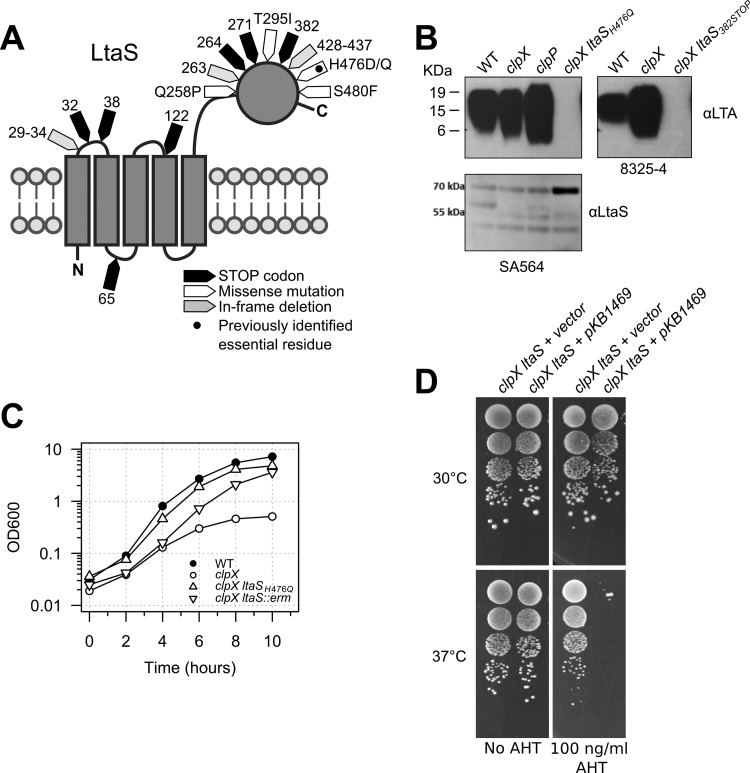
Mutations in the *ltaS* gene restore growth of the *S. aureus* clpX mutant. (A) Topology map of LtaS showing the location and class of the identified suppressor mutations. (B) Analysis of LTA synthesis. Extracts obtained from *S. aureus* strains SA564 (wild type), SA564 *clpX*, SA564 *clpP*, 564-25-2 (SA564 *clpX ltaS*_H476Q_), 8325-4 (wild type), 8325-4 *clpX*, and 83-25C (*clpX ltaS*_382STOP_) grown at 30°C and normalized based on optical density (OD_600_) readings of the original cultures were separated by SDS/PAGE, electrotransferred to a polyvinylidene difluoride (PVDF) membrane, and subjected to immunoblotting using an LTA-specific or an LtaS-specific antibody as indicated. (C) Growth of the *S. aureus* SA564 wild-type strain, the SA564 *clpX* strain, the 564-25-2 (SA564 *clpX ltaS*_H476Q_) spontaneous suppressor strain, and the SA564 *clpX ltaS*::*erm* strain at 30°C in TSB. (D) LtaS is essential at 37°C but not at 30°C. The 8325-4 *clpX ltaS*_382STOP_ suppressor mutant harboring either a plasmid expressing ClpX under the control of an AHT-inducible promoter or empty vector was grown in TSB at 37°C and diluted 10^1^-, 10^2^-, 10^3^-, and 10^4^-fold. Ten microliters of each dilution was spotted on TSA plates supplemented with AHT as indicated and subsequently incubated at either 30°C or 37°C.

When the *clpX* mutant strains were plated at 30°C, larger colonies consistently appeared among the very small colonies (Fig. 2A), indicating that the growth defect associated with lack of ClpX can be alleviated genetically by the acquisition of spontaneous suppressor mutations. The frequencies of large suppressor colonies in cultures of the *clpX* mutants after 24 h of growth at 30°C were 0.12 ± 0.06 in the 8325-4 *clpX* background and 0.92 ± 0.04 in the SA564 *clpX* background. One suspected suppressor mutant, strain 564-25-2, with restored growth at 30°C (Fig. 1A and 2B) was chosen for Illumina genome sequencing. The sequencing analysis revealed that the sequence of strain 564-25-2 deviated from that of the parental SA564 *clpX* strain by only one single nucleotide polymorphism (SNP) located in the *ltaS* gene and caused an H476Q substitution in the lipoteichoic acid (LTA) synthase enzyme, LtaS. Interestingly, the H476 residue was previously found to be essential for LtaS activity (18). To examine if mutations in *ltaS* were common among the *clpX* suppressor colonies, the *ltaS* gene of a number of the suppressor mutants was sequenced. The suppressor mutants were selected either directly on solid media without a culturing step or by passaging independent cultures of the 8325-4 *clpX* strain at 30°C until large suppressor colonies accounted for at least 90% of the CFU count (details are provided in Materials and Methods). Remarkably, mutations in *ltaS* were identified in 7 of the 10 independent 8325-4 *clpX* cultures as well as in a large number of the remaining suppressor strains selected in both the 8325-4 and SA564 strain backgrounds. As depicted in Fig. 3A, these mutations resulted in premature stop codons, amino acid substitutions, and in-frame deletions.

The *ltaS* mutations were predicted to be loss-of-function mutations based on the selection of a mutation changing the essential histidine at position 476 to a glutamine and on the frequent selection of mutations resulting in premature stop codons (18). Consistent with this prediction, the LTA polymer was absent from two representative strains, 83-25C (LtaS_382STOP_ isolated in the 8325-4 *clpX* background) and 564-25-2 (LtaS_H476Q_; Fig. 3B). The LTA immunoblot analysis showed a stronger LTA signal in the 8325-4 *clpX* mutant, indicating that ClpX impacts LTA expression; however, consistent with our experience that LTA immunoblots should be interpreted qualitatively rather than quantitatively, this difference was not reproducible (data not shown). LtaS expression was not altered by inactivation of either *clpX* or *clpP*, but a strong LtaS band was detected in strain 564-25-2, confirming previous results (18) that indicated that the nonfunctional LtaS_H476Q_ variant is expressed in larger amounts than the wild-type LtaS (Fig. 3B; see also Table S2 in the supplemental material).

The loss-of-function mutation in *ltaS* was the only genetic change introduced in the genome sequenced 564-25-2 suppressor mutant, strongly suggesting that inactivation of LtaS results in improved growth of the *clpX* mutant. In support thereof, the growth defect of the SA564 *clpX* strain was restored to a similar extent by disrupting the *ltaS* gene with an *ltaS*::*erm*-marked deletion introduced by phage transduction (Fig. 3C). We therefore conclude that inactivation of the conditionally essential LtaS synthase alleviates the growth defect caused by inactivation of the ClpX chaperone.

### LTA becomes nonessential in cells devoid of ClpX chaperone activity.

LTA has been reported to be essential for growth of *S. aureus* at 37°C. Therefore, we were surprised to find that the two *clpX* suppressor mutants confirmed to harbor loss-of-function mutations in *ltaS* grew better than the *clpX* single mutant, not only at 30°C but also at 37°C; the *clpX ltaS* double mutants were, however, not capable at growing at 44°C (Fig. 1A). The finding that the LTA-negative suppressor mutants grow well at 37°C indicates that LTA becomes nonessential in *S. aureus* cells lacking ClpX. To verify this, we performed a genetic linkage analysis to test whether the wild-type *clpX* allele (marked by inserting the *ermB* gene into a nearby noncoding sequence [19]) can be back-transduced into the *clpX ltaS* suppressor mutant. As shown in Table 1, the wild-type *clpX* gene was not cotransduced along with *ermB* into the LTA-negative *clpX* strains, whereas we obtained a high frequency of cotransduction using LTA-positive *clpX* mutant strains as recipients. This result is highly statistically significant (*P* < 0.0005), indicating that LTA is indeed essential under the conditions used in this study and that LtaS becomes nonessential in the absence of the ClpX chaperone. To confirm this result, plasmid pKB1469 expressing ClpX under control of an anhydrotetracycline (AHT)-inducible promoter was introduced into strain 83-25C. As predicted, this strain lost viability upon induction of ClpX expression at 37°C, showing that LtaS is essential in a ClpX-positive strain (Fig. 3D). In contrast, no effect on viability was observed when expression of ClpX was induced at 30°C (Fig. 3D), which is in line with the finding by Oku et al. (8) that LtaS is nonessential at 30°C.

**TABLE 1:** Numbers of transductants harboring either the wild-type or the Δ*clpX* allele determined using different *S. aureus* recipient strains

Cotransduction of wt *clpX* allele^a^	No. of phage transduction recipients for indicated strain				
	8325-4 *clpX*	8325-4 *clpX ltaS_382STOP_*	8325-4 *clpX ltaS_W122STOP_*	SA564 *clpX*	SA564 *clpX ltaS_H476Q_*
Yes	13	0	0	12	0
No	3	12	11	2	10

a Replacement of the Δ*clpX* allele with the wild-type (wt) allele was assessed by PCR.

### Inactivation of ClpX compensates for the absence of LTA by a novel c-di-AMP-independent pathway.

**FIG 4:**
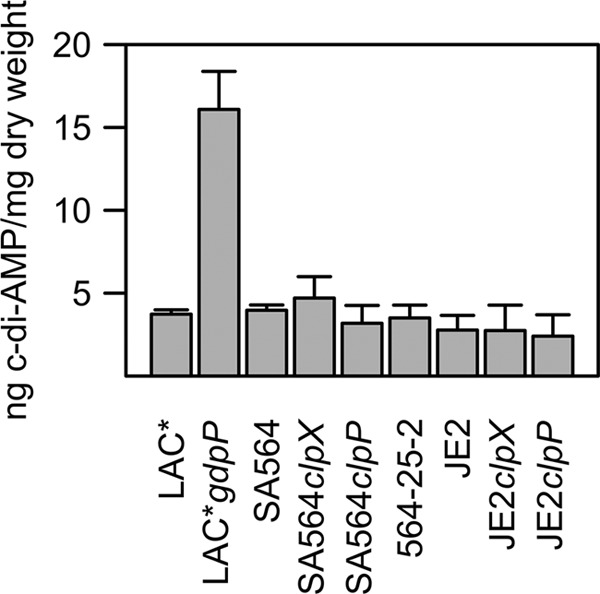
Cyclic-di-AMP levels in *S. aureus* are not affected by inactivation of *clpX* or *clpP*. Data represent intracellular c-di-AMP concentrations. The concentrations of c-di-AMP in the cytoplasm of strains LAC*, LAC* *gdpP*, SA564, SA564 *clpX*, SA564 *clpP*, 564-25-2 (*clpX ltaS*), JE2, JE2 *clpX*, and JE2 *clpP* were determined by LC-MS/MS. Values indicate c-di-AMP levels per milligram of bacterial dry weight, and the averages and standard deviations of results from three replicates are plotted.

An *S. aureus* gdpP mutant can, similarly to a *clpX* mutant, grow without LTA (7). The GdpP phosphodiesterase cleaves the second messenger c-di-AMP, and the intracellular concentration of c-di-AMP is consequently significantly increased in *gdpP* mutant strains (7). Interestingly, the *S. aureus* clpX mutant shares some phenotypes with a *gdpP* mutant, namely, a decrease in cell size, a small increase in peptidoglycan cross-linking (7, 19), and, as shown here, the ability to grow without LTA. To assess if these shared phenotypes correlated with increased intracellular c-di-AMP levels in the *clpX* mutant, we quantified c-di-AMP levels in cytoplasmic extracts from cultures of strains SA564 and JE2, the corresponding *clpX* mutant strains, and suppressor strain 564-25-2 (*ltaS clpX*) using a liquid chromatography-tandem mass spectrometry (LC-MS/MS) method (7). The results (Fig. 4) showed no significant differences between the wild types and the mutant strains in cellular levels of c-di-AMP, suggesting that inactivation of *clpX* bypasses the requirement for LTA by a c-di-AMP-independent pathway.

### Lack of ClpX improves septum formation in LTA-depleted cells.

**FIG 5:**
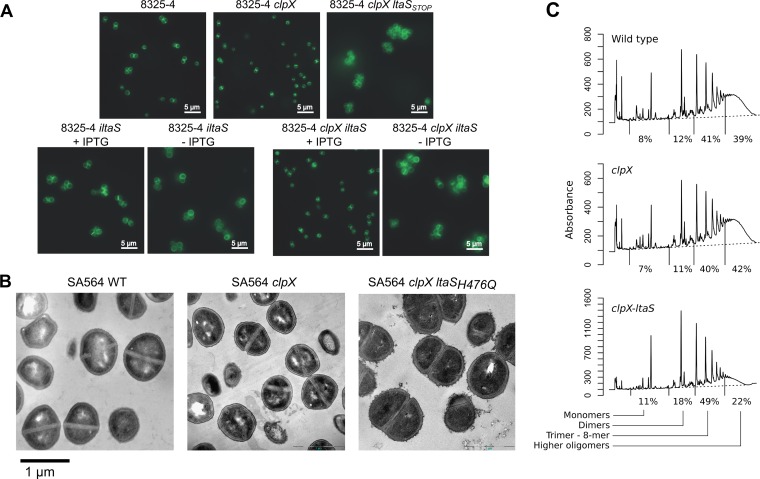
Cell morphology, septum formation, and cell wall structure are affected by inactivation of *clpX* and by the compensatory loss of LtaS activity. (A) Fluorescence microscopy analysis of wild-type, *clpX*-inactivated, and LTA-depleted strains. *S. aureus* strain 8325-4 and the corresponding *clpX* and *clpX-ltaS_STOP_* mutants were grown in TSB to exponential phase at 37°C. Strains 8325-4-*iltaS* and 8325-4 *clpX-iltaS* were grown to exponential phase at 37°C in the presence of 1 mM IPTG and then diluted into medium with or without 1 mM IPTG and grown for 3 h at 37°C. Bacteria were stained with BODIPY-vancomycin and prepared for fluorescence microscopy as described in Materials and Methods. Examples of raw microscope images can be seen in Fig. S4 to S7 in the supplemental material. (B) TEM analysis of wild-type, *clpX*-inactivated, and LTA-negative strains. The SA564 wild type, the SA564 *clpX* strain, and the 564-25-2 (SA564 *clpX ltaS*_H476Q_) strain were grown in TSB to log phase at 37°C and prepared for electron microscopy as described in Materials and Methods. (C) Peptidoglycan structure in the SA564 wild type, the SA564 *clpX* strain, and the 564-25-2 (SA564 *clpX ltaS*_H476Q_) strain. The figure panel shows HPLC chromatograms of mutanolysin-digested peptidoglycan purified from the indicated strains and percentages indicating the relative abundances of monomers, dimers, trimers to 8-mers, and higher oligomers in each strain. Results representative of two individual experiments are shown.

Depletion of LtaS in *S. aureus* confers a number of distinct phenotypes, including morphological changes such as an increase in cell size, misplacement of division septa, and a tendency to clump (6). Similar changes were reported when an RN4220 *ltaS* mutant was grown under conditions permissive for growth (8). Inactivation of *clpX*, on the other hand, did not impact septum localization but resulted in significantly smaller cells with a thickened cell wall (19). The finding that LtaS becomes nonessential in a *clpX* mutant strain prompted us to investigate if the absence of the ClpX chaperone counteracted some of the morphological phenotypes of LtaS-depleted cells. Light and transmission electron microscopy (TEM) of the wild type, the *clpX* single mutant, and the *clpX ltaS* double mutant grown at 30°C or 37°C showed that the majority of the *clpX ltaS* double mutant cells exhibited wild-type placement of the division septa, that is, septum formation that occurs at the midcell and mostly perpendicularly to previous division planes (Fig. 5A and B; see also Fig. S2 in the supplemental material). The *clpX ltaS* cells were, however, larger and more irregularly shaped than the wild-type cells or *clpX* single mutant cells, and, additionally, the cell wall was thicker and more irregular and had a fuzzier appearance (Fig. 5B). To directly investigate the effect on cell division of depleting LTA in cells that lack *clpX*, *S. aureus* strains with IPTG (isopropyl-β-d-thiogalactopyranoside)-inducible *ltaS* expression were created in either an 8325-4 wild-type (8325-4-*iltaS*) or an 8325-4 *clpX* (8325-4-*clpX-iltaS*) genetic background. As expected, in the absence of IPTG and, hence, of *ltaS* expression, cells with an intact *clpX* gene stopped growing whereas cells with a disrupted *clpX* gene were able to continue growth, corroborating the conclusion stated above that LTA becomes nonessential when ClpX is inactivated (see Fig. S3). Extensive cell wall synthesis at the division site in the presence of LTA was observed both in the presence and the absence of an intact *clpX* gene (Fig. 5A; see also Fig. S4 and S5). Depletion of LTA also resulted in cell enlargement regardless of *clpX* status (Fig. 5A; see also Fig. S4 and S5). However, while LTA depletion led to a reduction in visible septum formation in the wild-type background, septum formation was still prevalent after LTA depletion in the *clpX* background (Fig. 5A; see also Fig. S6 and S7). In summary, inactivation of *clpX* appeared to rescue the reduced ability of LTA-deficient cells to lay down the septum correctly, whereas lack of *clpX* did not counteract other phenotypes associated with the lack of LTA: namely, larger cells, a thicker and more irregular cell wall, and cell clustering.

### Inactivation of LtaS decreases peptidoglycan cross-linking and increases susceptibility to vancomycin and cefoxitin.

Inactivation of *clpX* causes a small increase in peptidoglycan cross-linking and decreases susceptibility to several types of antibiotics targeting the cell wall; hence, we speculated that the cellular changes underlying these phenotypes might be correlated with the ability of the *clpX* mutant to tolerate LTA deficiency (19). To examine the changes in cell wall properties in more detail, we compared the muropeptide profiles of SA564 wild-type, *clpX*, and *clpX ltaS* mutant cells. This analysis revealed that while the peptidoglycan profiles of SA564 wild-type and *clpX* mutant cells are very similar, the peptidoglycan from the LTA-deficient *clpX ltaS* mutant was characterized by a severe reduction in the levels of highly cross-linked muropeptides (>8-mers) and a concomitant increase in the ratio of dimers to octamers (Fig. 5C). Next, we determined the susceptibilities of the strains toward antibiotics targeting the cell envelope. This analysis revealed that the *clpX ltaS* double mutant is more sensitive toward cefoxitin and vancomycin than the *clpX* mutant and the wild-type strains and is slightly more sensitive to oxacillin (Table 2). In contrast, the *ltaS* mutation did not affect susceptibility to imipenem and ertapenem, both of which belong to the carbapenem class of β-lactams. As shown previously (19), the *clpX* deletion had only minor effects on the susceptibility of strains SA564 and 8325-4 to oxacillin and daptomycin and no effect on susceptibility to cefoxitin and vancomycin. Taken together, these results indicate that the introduction of loss-of-function mutations in *ltaS* concomitantly decreased peptidoglycan cross-linking and susceptibility to some cell wall-targeting antibiotics. The effect of the *clpX* and *ltaS* mutations varied greatly with the type of antibiotic, and there was no trend supporting the notion that the cellular changes responsible for decreasing the antibiotic susceptibility of the *clpX* mutant were contributing to the ability of the *clpX* mutant to grow in the absence of LTA.

**TABLE 2:** MICs of different antibiotics for the SA564 and 8325-4 wild types and *clpX* and *clpX ltaS* mutants

Antibiotic	MIC (mg/liter)					
	8325-4			SA564		
	wt	*clpX*	*clpX ltaS_382STOP_*	wt	*clpX*	*clpX ltaS_H476Q_*
Oxacillin	0.19–0.25	0.38–0.5	0.094	0.38–0.5	1.5–4	0.25
Imipenem	0.047	0.047	0.032	0.064	0.064	0.023
Ertapenem	0.19	0.19	0.094	0.19	0.25	0.19
Cefoxitin	3	3	0.38	4	4	1
Daptomycin	0.38–0.75	0.75–1	0.38	0.25	0.38	0.38
Vancomycin	1.5	1.5	0.25	1.5	1	0.19

### LTA and ClpX have opposite roles in determining the level of surface-bound autolysins.

**FIG 6:**
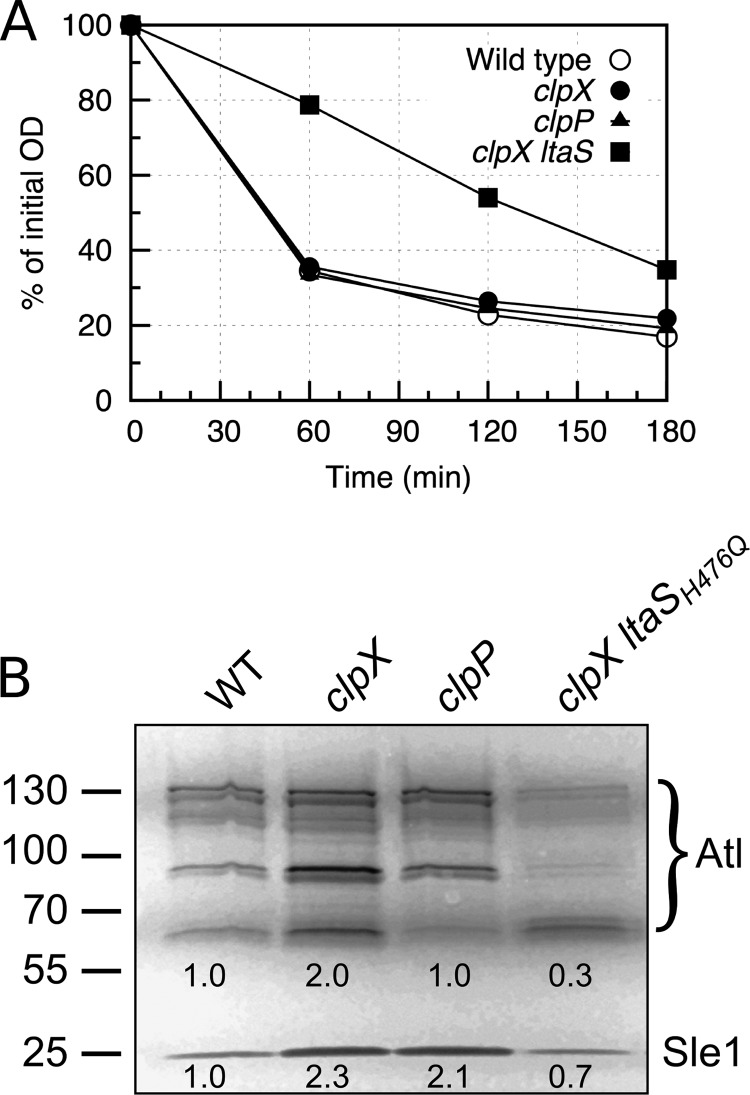
An LTA-negative strain has decreased autolytic activity. (A) Autolysis assay. *S. aureus* strain SA564 and the corresponding *clpX*, *clpP*, and *clpX ltaS_H476Q_* mutants were analyzed for autolytic activity using a Triton X-100 autolysis assay. (B) Zymogram assay. SA564 and its derived strains were grown to exponential phase, and equal amounts of cell wall-associated proteins were loaded onto an SDS polyacrylamide gel containing heat-killed *S. aureus* SA564 wild-type cells. Results representative of three independent experiments are shown. The positions of the molecular mass standards are indicated (in kilodaltons) on the left. Atl and Sle1 protein levels were quantified relative to the levels seen with the wild-type strain using ImageJ.

LTA is critical for maintaining normal levels of peptidoglycan hydrolase activity, and binding of Atl, the major *S. aureus* autolysin, to the cell wall seems to involve direct interactions between Atl and LTA (20). We therefore examined the autolytic activity of our strains and found that the autolytic activity of the *clpX ltaS* double mutant was greatly reduced in a Triton X-100 autolysis assay, demonstrating that the *clpX ltaS* double mutant, similarly to LTA-depleted cells, had reduced autolytic activity (Fig. 6A). To follow up on this finding, a zymographic analysis was performed. In the zymographic analysis, the activity of the major autolysin, Atl, was visible in multiple bands, reflecting the fact that Atl is a bifunctional murein hydrolase that is produced as a 138-kDa precursor protein, sequentially cleaved to generate 115- and 85-kDa intermediate products, and further processed to generate a 62-kDa *N*-acetylmuramyl-l-alanine amidase (AM) and a 51-kDa N-acetylglucosaminidase (GL; Fig. 6B). Consistent with the Triton-X-autolysis assay, the zymographic profile of cell extracts derived from the *clpX ltaS* double mutant demonstrated that the intensity of Atl bands in the *clpX ltaS* double mutant was reduced compared to the profiles of the wild-type or *clpX* mutant cells. The only exception was the band originating from the 62-kDa mature AM, which had the same intensity as in wild-type extracts. In contrast, inactivation of *clpX* alone increased the intensity of this band and of an 85-kDa variant of Atl (Fig. 6B). In the zymographic analysis, the activity of the Sle1 autolysin was also clearly visible. As previously reported, Sle1 is a substrate of ClpXP and consequently present at elevated levels in *clpX* and *clpP* mutant cells (21). Strikingly, the level of Sle1, similarly to the 62-kDa mature AM, was again reduced to wild-type levels in the *clpX ltaS* double mutant.

*S. aureus* encodes a number of additional enzymes predicted to possess autolytic activity, but their activity was not visible in the zymographic analysis. In order to investigate the general impact of ClpX and LTA on the levels of autolytic enzymes bound to the cell surface, the surface proteome (“surfacome”) was analyzed by trypsin shaving followed by MS identification. The surfacome analyses identified two other predicted autolysins, SceD and Aaa, in addition to Atl that were identified with the highest identification scores in the surfacome of the single *clpX* mutant strain (see Table S2 in the supplemental material), suggesting that the three autolysins are expressed at higher levels or that their stability is increased at the cell surface of the *clpX* mutant compared to the wild-type or the *clpX ltaS* strain. This confirms that ClpX has a negative impact on the levels of Atl and two other surface-bound autolysins. The comparable identification scores of these three autolysins shaved from wild-type and *clpX ltaS* double mutant cells further support the notion that inactivation of LtaS in cells lacking ClpX causes the amounts of these surface-bound autolysins to revert to wild-type levels. Taken together, our results suggest that LTA and ClpX have opposite roles in determining the level of several surface-bound autolysins.

## DISCUSSION

LTA is an abundant cell wall polymer that has attracted interest as a novel antibacterial target owing to its essential role in growth of *S. aureus* (22). Moreover, LTA seems to have an important role in resistance to β-lactams, as MRSA strains can be resensitized to some β-lactams by reducing expression of the *ltaS* gene (23). Thus, compounds targeting LTA synthesis may have the additional advantage that they can be used in combination with β-lactams to effectively combat MRSA.

The exact role of LTA remains unknown, and the pleiotropic phenotypes associated with depletion of LTA have represented a challenge for dissecting why LTA is conditionally essential for growth of *S. aureus*. In the present study, we showed that mutations abolishing the function of LtaS arise spontaneously in an *S. aureus* mutant lacking the ClpX chaperone. The finding that the combined deletion of the *ltaS* and *clpX* genes results in cells that grow almost as well as wild-type cells, despite the fact that each of the single deletions confers a severe growth defect to *S. aureus* cells, indicates that ClpX and LTA have opposite roles in a pathway that is critical for growth of *S. aureus*. The effect of deleting either the *clpX* or the *ltaS* gene is strongly dependent on temperature: deletion of *clpX* impairs growth severely at 30°C and mildly at 37°C, and, in contrast, deletion of *ltaS* results in loss of viability at 37°C but, for some strains, not at 30°C.

Phenotypic analysis revealed that the *clpX ltaS* double mutant retained most of the hallmark phenotypes associated with LTA depletion, including morphological changes such as enlarged cells, a thickened, irregular cell wall, and impaired cell separation following cell division. The latter phenotype has been explained by the substantially reduced autolytic activity in cells lacking LTA (8). Interestingly, we showed here that a number of autolysins, including the major autolysin Atl, are more abundant in the surfacome of *S. aureus* cells lacking ClpX and that the amount of autolysins is reduced to the wild-type level or lower by the subsequent inactivation of LtaS. Taken together, our results show that LTA and ClpX have opposite roles in determining the level of surface-bound autolysins. Hence, one possible scenario is that the dual inactivation of ClpX and LtaS may help to balance the aberrant autolytic activity caused by the inactivation of either ClpX or LtaS alone. However, the validity of this hypothesis is questioned by our finding that the *clpX ltaS* double mutant, despite the nearly wild-type expression of Atl and other autolysins, retained phenotypes associated with reduced expression of autolysins such as a deficiency in cell separation and very low autolytic activity in a Triton X-100 autolysis assay.

LTA depletion is also characterized by reduced cell wall synthesis at the septum and aberrant positioning of division septa (7, 8). Intriguingly, we showed here that in LTA-depleted cells that lack a functional *clpX* gene, extensive septum formation was observed at midcell, suggesting that inactivation of *clpX* alleviates to some extent the septum placement defects associated with LTA deficiency. The partly restored septum formation in cells lacking both ClpX and LTA implies that cell division is central to understanding the functional link between ClpX activity and LTA activity. In almost all bacteria, cell division is orchestrated by the FtsZ tubulin homologue, which polymerizes to form a ring-like structure, the Z-ring, which establishes the location of the division site (24). The Z-ring is tethered to the cell membrane by the essential FtsA protein, which also has a role in the formation of larger FtsZ bundles, as well as in recruiting downstream cell division proteins (24). It has been found that the ClpX chaperone modulates FtsZ polymer dynamics in bacteria as diverse as *Escherichia coli*, *B. subtilis*, and *Mycobacterium tuberculosis*, suggesting that ClpX, interacting directly with FtsZ, has a widely conserved role in cell division (25–27). Similarly, several observations indicated that LtaS also has a direct role in cell division. First, LtaS accumulates at the division site, where it has been proposed to be part of a large multienzyme complex containing early-stage division proteins such as FtsA, as well as late-stage cell division proteins (9). Additionally, *S. aureus* cells depleted for both LTA and WTA lose the ability to establish the Z-ring (28). The literature therefore supports the scenario that both LTA and ClpX have direct roles in cell division. Our finding that the combined inactivation of LtaS and ClpX abolished the growth defect conferred by each of the single mutations suggests that ClpX and LTA have antagonistic roles in the cell division process of *S. aureus*.

In conclusion, we have shown that the LTA cell wall polymer becomes nonessential for growth of *S. aureus* under conditions in which cells are devoid of ClpX chaperone activity as a consequence of the activity of a novel c-di-AMP-independent pathway. The presented data indicate that ClpX and LTA have opposing roles in the coordination of two key *S. aureus* cell division processes, namely, septum formation and control of autolytic activity, thereby providing novel insight into the function of this important cell wall polymer of Gram-positive bacteria.

## MATERIALS AND METHODS

### Bacterial strains and growth conditions.

Strains used in this study are listed in Table S1 in the supplemental material. Strain construction details are described in Text S1 in the supplemental material. *E. coli* strains were cultured in Luria broth (LB) at 37°C or 30°C or on LB medium solidified with 1.5% (wt/vol) agar. *S. aureus* strains were cultured in tryptic soy broth (TSB) at 37°C or 30°C with aeration or on TSB medium solidified with 1.5% (wt/vol) agar (TSA). In inoculating *clpX* deletion strains, care was taken to avoid visibly larger colonies containing potential suppressor mutants. *S. aureus* JE2-derived strains were obtained from the Network of Antimicrobial Resistance in *Staphylococcus aureus* (NARSA) program (supported by NIAID/NIH contract HHSN272200700055C).

### Growth curves and growth rate calculations.

Growth of *S. aureus* strains was assessed by measuring the optical density at 600 nm (OD_600_) of cultures grown in Erlenmeyer flasks or in a Bioscreen C instrument (Oy Growth Curves Ab Ltd., Finland) and by determining the number of CFU per milliliter in cultures grown in Erlenmeyer flasks. Overnight cultures were grown in TSB at 37°C. For determination of growth curves in Erlenmeyer flasks, overnight cultures were diluted 1:200 in 30 ml TSB, giving a starting OD_600_ of approximately 0.015 to 0.03, and were incubated with aeration at 30°C or 37°C with or without antibiotics as indicated. OD_600_ values were determined at 1-h intervals, and CFU counts per milliliter culture were determined at 2-h intervals by performing serial dilutions in 0.9% (wt/vol) NaCl and plating 100 µl on TSB plates. Plates were then incubated at 37°C, and colonies were enumerated after overnight growth. The colony size distribution in *clpX* cultures was clearly bimodal, allowing easy differentiation of smaller and larger (presumed suppressor) colonies. For growth in the Bioscreen C instrument, overnight cultures were diluted in 300 µl TSB to an OD_600_ of approximately 0.001, and the OD_600_ was measured every 5 min with 20 s of shaking before each measurement.

All exponential growth rates were determined by growing the relevant strains in a Bioscreen C instrument as described above. The growth rates were automatically calculated by applying a custom R script (29) using a linear regression function kindly provided by the Botstein laboratory (http://www.princeton.edu/genomics/botstein/protocols/Growth-Rate-Using-R.pdf). In short, OD_600_ values were log-transformed and linear regressions were determined for each data point in the OD_600_ interval from 0.02 to 0.12 based on a window containing 15 data points. The exponential growth rate was identified as the maximal slope of the linear regressions. The script for calculation of growth rates was validated using simulated growth data obtained from the logistic growth equation for growth yield as a function of time (t), growth rate (μ), and lag time (30). Standard deviations were calculated using values from different biological replicates. Statistical significance was calculated using Student’s *t* test. Doubling time in minutes was calculated as ln 2/μ × 60.

### Genetic suppressor analysis.

SA564 and 8325-4 *clpX* mutants were streaked for single colonies on tryptic soy agar plates and incubated at 37°C overnight. Several small colonies were then resuspended in 0.9% (wt/vol) NaCl, and dilutions were spread on TSA plates that were incubated at 25°C, 30°C, or 37°C. Colonies that appeared visibly larger than the background colonies were colony purified twice and stored. In addition, ten independent overnight cultures of 8325-4 *clpX* grown at 37° were diluted 1:1,000 into 1 ml fresh TSB medium and incubated with aeration at 30°C. After 24 h, the cultures were transferred again to fresh medium by a 1:1,000 dilution. Each day, bacteria were plated on TSA at 30°C to determine the proportion of large suppressor colonies. After two passages at 30°C, all cultures contained at least 90% suppressors, and one large colony from each culture was purified and stored. The *ltaS* gene was amplified by PCR using primers KB71F and KB71R and genomic DNA prepared from 26 suppressor mutants isolated in the SA564 and 8325-4 backgrounds and sequenced by capillary sequencing using primers KB71F, KB73F, and KB71R.

### Cotransduction experiment.

KB1241 was used as the donor for bacteriophage Φ11 transduction into strains 8325-4Δ*clpX*, 83-25C, and KB1262 or for bacteriophage Φ85 transduction into strains SA564Δ*clpX* and 564-25-2, selecting for erythromycin (5 mg/liter) resistance. The presence of the *ermB* gene was confirmed in the transductants by PCR using primers KB76F and KB76R (19) prior to testing for cotransduction of the wild-type *clpX* allele by PCR with primers saclpx385f and saclpx2447r (14). Statistical significance was calculated using Pearson’s chi-squared test.

### SNP detection.

One of the suppressor mutants obtained in the SA564 *clpX* genetic background (564-25-2) was chosen for SNP detection. Genomic DNA was prepared from overnight cultures of the SA564 *clpX* strain and the 564-25-2 suppressor strain grown in TSB at 37°C and was sequenced on an Illumina Hi-Seq 2000 instrument (Fasteris, SA, Geneva, Switzerland). The sequencing reads obtained by Illumina sequencing of SA564 *clpX* and the 564-25-2 suppressor mutant were aligned to the assembled SA564 genome (GenBank accession numbers CP010890 and CP010891 for chromosome and plasmid, respectively [31]) using Bowtie2 (version 2.2.3, default parameters [32]). The sequences have been submitted to the European Nucleotide Archive (see below for accession number). Alignments were converted to sorted, indexed binary alignment map (BAM) files, and SNPs and indels were called with SAMtools and BCFtools (version 1.1, default parameters [33]). Using BEDtools (version 2.21.0 [34]), the average read coverage was calculated to a total of 695 for strain 564-25-2 and a total of 410 for strain SA564 Δ*clpX*. The sequencing reads from SA564 *clpX* or 564-25-2 covered the whole reference genome except for the expected *clpX* deletion in both strains (position 2066206 to position 2066840) and a 14-nt deletion in an intergenic region (position 2273962 to position 2273975) in both strains. The SA564 reference genome was annotated with the RAST server (35), and annotations and calculated amino acid changes were assigned to the variant set using a custom R script. Variants were excluded if the quality score for the base call was <100 or if the Phred-scaled *P* value using the Fisher exact test for strand bias was >200 for indels or >60 for SNPs. The filtered variants were verified by capillary sequencing using appropriate PCR primers (see Table S2 in the supplemental material). By this method, one SNP (A2274642T) in the tRNA-Leu-CAA gene was identified between the reference genome and our SA564 wild type, no SNPs were identified between the SA564 wild type and the SA564 *clpX* mutant, and one SNP (C1082972A) in the *ltaS* gene (LtaS_H476Q_) was identified between strain 564-25-2 and the SA564 *clpX* mutant.

### LtaS depletion and fluorescence microscopy.

*S. aureus* strains 8325-4, 8325-4-Δ*clpX*, and 8325-4-Δ*clpXltaS_stop_* were grown overnight at 37°C in TSB. Strains 8325-4-*iltaS* and 8325-4Δ*clpX-iltaS* were grown in TSB with erythromycin (10 µg/ml) and 1 mM IPTG. The following day, bacteria from a 1-ml culture aliquot were harvested and washed three times with 1 ml TSB. Next, 6 ml TSB (supplemented where necessary with 1 mM IPTG) was inoculated with 60 µl washed bacterial suspensions (1:100 dilution) and cultures were incubated at 37°C with shaking. The OD_600_ values were recorded every 2 h for 8 h. To maintain the culture in log phase, at the 4-h time point, the cultures were again back diluted 1:100 into 5 ml fresh TSB (supplemented when required with 1 mM IPTG) and growth continued for an additional 4 h. The average OD_600_ values and standard deviations from three independent experiments were plotted. At the 4-h time point, 1 ml of culture was removed and prepared for the detection of LTA by Western blotting.

For microscopy, strains were grown as described above with the exception that strain NCTC8325-4-*iltaS* propagated in the absence of IPTG was diluted 1:10 at the 4-h point to obtain sufficient material for the subsequent microscopy analysis. At the 7-h time point, bacteria from 2 ml of culture were harvested by centrifugation for 2 min at 17,000 × *g*. Cells were washed 3 times with phosphate-buffered saline (PBS) (pH 7.4). The cells were suspended in a volume of PBS to give a final OD_600_ of 3. Coverslips were prepared in advance by coating them with 100 µl of a 0.1% polylysine solution and washing 4 times with PBS (pH 7.4). Next, 100 µl of cells was placed on the coverslip for 3 min and washed 3 times with PBS. Finally, 100 µl of a 1 µg/ml vancomycin–boron-dipyrromethene (BODIPY) (Molecular Probes) solution was added to the immobilized bacteria, and the bacteria were incubated for 5 to 20 min and subsequently washed 3 times with PBS. The coverslips were mounted onto a slide containing 20 µl of Vectashield (Vector Laboratories). Cells were visualized on a Zeiss Axio Imager A2 microscope, using a green fluorescent protein (GFP) filter, and images were captured with an AxioCam MRc Rev.3 camera.

### Electron microscopy.

Overnight cultures grown at 37°C were diluted 1:200 into 40 ml of fresh TSB and grown at 30°C or 37°C to an OD_600_ of 0.5. Bacteria from a 10-ml culture aliquot were collected by centrifugation at 8,000 × *g*, and the cell pellets were suspended in fixation solution (2.5% glutaraldehyde–0.1 M cacodylate buffer [pH 7.4]; Electron Microscopy Sciences, PA) and incubated overnight at 4°C. The fixed cells were further treated with 2% osmium tetroxide, followed by 0.25% uranyl acetate for contrast enhancement. The pellets were dehydrated in increasing concentrations of ethanol, followed by pure propylene oxide, and were then embedded in Epon resin. Thin sections for electron microscopy were stained with lead citrate and observed using a Philips CM100 BioTWIN transmission electron microscope fitted with an Olympus Veleta camera with a resolution of 2,048 by 2,048 pixels. Sample processing and microscopy were performed at the Center for Integrated Microscopy, Faculty of Health and Medical Sciences, University of Copenhagen.

### Peptidoglycan analysis.

One liter (for the wild type and the 564-25-2 mutant) or 2 liters (for the *clpX* mutant) of TSB medium was inoculated with overnight cultures of *S. aureus* strains SA564 and SA564Δ*clpX* and strain 564-25-2 to an OD_600_ of 0.06 and grown at 30°C to late-log phase (OD of approximately 1.6 for the wild type and strain 564-25-2 and 0.8 for the *clpX* strain). The cultures were cooled on ice, and the bacteria were subsequently collected by centrifugation. Peptidoglycan was purified, digested with mutanolysin, and analyzed by high-performance liquid chromatography (HPLC), as described previously (7). Muropeptide profiles were determined for each strain in duplicate, and HPLC chromatograms from one representative experiment are shown. Data analysis was carried out as described previously (19).

### Accession number.

The sequences obtained by Illumina sequencing of SA564 *clpX* and the 564-25-2 suppressor mutant have been submitted to the European Nucleotide Archive under accession number PRJEB14861.

## SUPPLEMENTAL MATERIAL

Figure S1  Effect of size and growth phase of inoculum on growth of *clpX* mutant. *S. aureus* strains 8325-4 and 8325-4 *clpX* were grown to exponential (OD, ~0.1; “exp”) or stationary (overnight culture; “stat”) phase at 37°C and diluted (1:10 or 1:1,000) as indicated into 300 µl TSB, and growth was measured at 30°C in a Bioscreen C instrument. Values represent mean OD readings ± standard deviations (error bars are smaller than the symbols) of the results from three biological replicates. Download Figure S1, TIF file, 1.1 MB

Figure S2  Microscopic analysis of the wild-type, *clpX*-inactivated, and LTA-negative strains. The *S. aureus* SA564 wild type, the SA564 *clpX* strain, and the 564-25-2 (SA564 *clpX ltaS_H476Q_*) strain were grown in TSB to the log phase at either 30°C or 37°C, samples were prepared for microscopy analysis, and bacteria were stained with vancomycin-BODIPY. Left panels show phase-contrast images; right panels show fluorescence images. Download Figure S2, TIF file, 1.5 MB

Figure S3  (A) Growth curves of *S. aureus* 8325-4 and derived strains. Overnight cultures of *S. aureus* strains 8325-4 (WT), 8325-4-Δ*clpX*, 8325-4-Δ*clpX ltaS_stop_*, 8325-4-*iltaS* (WT-*iltaS*), and 8325-4Δ*clpX-iltaS* were harvested, washed, and diluted 1:1,000 into TSB substituted with 1 mM IPTG. Cultures were incubated at 37°C, and OD_600_ values were recorded. To maintain the cultures in log phase, the cultures were back diluted 1:100 at the 4-h time point into 5 ml fresh TSB (supplemented when required with 1 mM IPTG) and growth continued for an additional 4 h. The average OD_600_ values and standard deviations from three independent experiments are plotted. (B) Analysis of LTA synthesis. Extracts obtained from *S. aureus* strains 8325-4 (WT), 8325-4-Δ*clpX*, 8325-4-Δ*clpX ltaS_stop_*, 8325-4-*iltaS* (WT-*iltaS*), and 8325-4Δ*clpX-iltaS* were separated by SDS/PAGE, electrotransferred to a PVDF membrane, and subjected to immunoblotting using an LTA-specific antibody. Download Figure S3, TIF file, 0.7 MB

Figure S4  Fluorescence microscopic analysis of strain 8325-4-*iltaS* grown to exponential phase at 37°C in the presence of 1 mM IPTG, and stained with BODIPY-vancomycin. Bacteria were grown and prepared for fluorescence microscopy as described in Materials and Methods. Download Figure S4, TIF file, 1 MB

Figure S5  Fluorescence microscopic analysis of strain 8325-4-*clpX-iltaS* grown to exponential phase at 37°C in the presence of 1 mM IPTG and stained with BODIPY-vancomycin. Bacteria were grown and prepared for fluorescence microscopy as described in Materials and Methods. Download Figure S5, TIF file, 1.2 MB

Figure S6  Fluorescence microscopic analysis of strain 8325-4-*iltaS* grown to exponential phase at 37°C in the absence of IPTG and stained with BODIPY-vancomycin. Bacteria were grown and prepared for fluorescence microscopy as described in Materials and Methods. Download Figure S6, TIF file, 0.9 MB

Figure S7  Fluorescence microscopic analysis of strain 8325-4-*clpX-iltaS* grown to exponential phase at 37°C in the absence of IPTG and stained with BODIPY-vancomycin. Bacteria were grown and prepared for fluorescence microscopy as described in Materials and Methods. Download Figure S7, TIF file, 0.8 MB

Table S1  Bacterial strains, plasmids, and primers used in this study. Table S1, PDF file, 0.1 MB

Table S2  List of all identified surface-associated proteins from SA564 and its mutant derivatives (strains *ΔclpX* and *ΔclpX-ltsA*) cultured at 30°C or 37°C. All trypsin digestions were conducted with duplicate cells. Relevant information related to protein functions, physicochemical characteristics, and subcellular location are described in Text S1. EC, extracellular; CP, cytoplasmic; CP/M, cytoplasmic/membrane; CW, cell wall; [pg], paragon; [ms], mascot. No. matc. pept, number of matched peptides. Cells shown in red and green refer to identification scores ([pg], [ms]) suggesting higher or lower protein abundances in the mutant strains than in the parental SA564 strain. Cells shown in gray indicate that proteins were not present in detectable amounts. Table S2, XLSX file, 0.04 MB

Text S1  Supplementary Materials and Methods. Download Text S1, PDF file, 0.1 MB

## References

[1] Lowy FD 1998 *Staphylococcus aureus* infections. N Engl J Med 339:520–532. doi:10.1056/NEJM199808203390806. 9709046

[2] DeLeo FR, Otto M, Kreiswirth BN, Chambers HF 2010 Community-associated meticillin-resistant *Staphylococcus aureus*. Lancet 375:1557–1568. doi:10.1016/S0140-6736(09)61999-1. 20206987 PMC3511788

[3] Richter SG, Elli D, Kim HK, Hendrickx AP, Sorg JA, Schneewind O, Missiakas D 2013 Small molecule inhibitor of lipoteichoic acid synthesis is an antibiotic for Gram-positive bacteria. Proc Natl Acad Sci U S A 110:3531–3536. doi:10.1073/pnas.1217337110. 23401520 PMC3587227

[4] Percy MG, Gründling A 2014 Lipoteichoic acid synthesis and function in gram-positive bacteria. Annu Rev Microbiol 68:81–100. doi:10.1146/annurev-micro-091213-112949. 24819367

[5] Schneewind O, Missiakas D 2014 Lipoteichoic acids, phosphate-containing polymers in the envelope of gram-positive bacteria. J Bacteriol 196:1133–1142. doi:10.1128/JB.01155-13. 24415723 PMC3957714

[6] Gründling A, Schneewind O 2007 Synthesis of glycerol phosphate lipoteichoic acid in *Staphylococcus aureus*. Proc Natl Acad Sci U S A 104:8478–8483. doi:10.1073/pnas.0701821104. 17483484 PMC1895975

[7] Corrigan RM, Abbott JC, Burhenne H, Kaever V, Gründling A 2011 c-di-AMP is a new second messenger in *Staphylococcus aureus* with a role in controlling cell size and envelope stress. PLoS Pathog 7:e1002217. doi:10.1371/journal.ppat.1002217. 21909268 PMC3164647

[8] Oku Y, Kurokawa K, Matsuo M, Yamada S, Lee B-L, Sekimizu K 2009 Pleiotropic roles of polyglycerolphosphate synthase of lipoteichoic acid in growth of *Staphylococcus aureus* cells. J Bacteriol 191:141–151. doi:10.1128/JB.01221-08. 18952789 PMC2612411

[9] Reichmann NT, Piçarra Cassona C, Monteiro JM, Bottomley AL, Corrigan RM, Foster SJ, Pinho MG, Gründling A 2014 Differential localization of LTA synthesis proteins and their interaction with the cell division machinery in *Staphylococcus aureus*. Mol Microbiol 92:273–286. doi:10.1111/mmi.12551. 24533796 PMC4065355

[10] Schirner K, Marles-Wright J, Lewis RJ, Errington J 2009 Distinct and essential morphogenic functions for wall- and lipo-teichoic acids in *Bacillus subtilis*. EMBO J 28:830–842. doi:10.1038/emboj.2009.25. 19229300 PMC2670855

[11] Webb AJ, Karatsa-Dodgson M, Gründling A 2009 Two-enzyme systems for glycolipid and polyglycerolphosphate lipoteichoic acid synthesis in Listeria monocytogenes. Mol Microbiol 74:299–314. doi:10.1111/j.1365-2958.2009.06829.x. 19682249 PMC2764115

[12] Kirstein J, Hoffmann A, Lilie H, Schmidt R, Rübsamen-Waigmann H, Brötz-Oesterhelt H, Mogk A, Turgay K 2009 The antibiotic ADEP reprogrammes ClpP, switching it from a regulated to an uncontrolled protease. EMBO Mol Med 1:37–49. doi:10.1002/emmm.200900002. 20049702 PMC3378108

[13] Farrand AJ, Reniere ML, Ingmer H, Frees D, Skaar EP 2013 Regulation of host hemoglobin binding by the *Staphylococcus aureus* Clp proteolytic system. J Bacteriol 195:5041–5050. doi:10.1128/JB.00505-13. 23995637 PMC3811588

[14] Frees D, Qazi SN, Hill PJ, Ingmer H 2003 Alternative roles of ClpX and ClpP in *Staphylococcus aureus* stress tolerance and virulence. Mol Microbiol 48:1565–1578. doi:10.1046/j.1365-2958.2003.03524.x. 12791139

[15] Frees D, Sørensen K, Ingmer H 2005 Global virulence regulation in *Staphylococcus aureus*: pinpointing the roles of ClpP and ClpX in the sar/agr regulatory network. Infect Immun 73:8100–8108. doi:10.1128/IAI.73.12.8100-8108.2005. 16299304 PMC1307069

[16] Jelsbak L, Ingmer H, Valihrach L, Cohn MT, Christiansen MH, Kallipolitis BH, Frees D 2010 The chaperone ClpX stimulates expression of *Staphylococcus aureus* protein A by rot dependent and independent pathways. PLoS One 5:e12752. doi:10.1371/journal.pone.0012752. 20856878 PMC2939077

[17] Kullik I, Giachino P, Fuchs T 1998 Deletion of the alternative sigma factor sigmaB in *Staphylococcus aureus* reveals its function as a global regulator of virulence genes. J Bacteriol 180:4814–4820. 9733682 10.1128/jb.180.18.4814-4820.1998 PMC107504

[18] Lu D, Wörmann ME, Zhang X, Schneewind O, Gründling A, Freemont PS 2009 Structure-based mechanism of lipoteichoic acid synthesis by *Staphylococcus aureus* LtaS. Proc Natl Acad Sci U S A 106:1584–1589. doi:10.1073/pnas.0809020106. 19168632 PMC2635763

[19] Bæk KT, Gründling A, Mogensen RG, Thøgersen L, Petersen A, Paulander W, Frees D 2014 β-Lactam resistance in methicillin-resistant *Staphylococcus aureus* USA300 is increased by inactivation of the ClpXP protease. Antimicrob Agents Chemother 58:4593–4603. doi:10.1128/AAC.02802-14. 24867990 PMC4136064

[20] Zoll S, Schlag M, Shkumatov AV, Rautenberg M, Svergun DI, Götz F, Stehle T 2012 Ligand-binding properties and conformational dynamics of autolysin repeat domains in staphylococcal cell wall recognition. J Bacteriol 194:3789–3802. doi:10.1128/JB.00331-12. 22609916 PMC3416534

[21] Feng J, Michalik S, Varming AN, Andersen JH, Albrecht D, Jelsbak L, Krieger S, Ohlsen K, Hecker M, Gerth U, Ingmer H, Frees D 2013 Trapping and proteomic identification of cellular substrates of the ClpP protease in *Staphylococcus aureus*. J Proteome Res 12:547–558. doi:10.1021/pr300394r. 23253041

[22] Pasquina LW, Santa Maria JP, Walker S 2013 Teichoic acid biosynthesis as an antibiotic target. Curr Opin Microbiol 16:531–537. doi:10.1016/j.mib.2013.06.014. 23916223 PMC3834221

[23] Meredith TC, Wang H, Beaulieu P, Gründling A, Roemer T 2012 Harnessing the power of transposon mutagenesis for antibacterial target identification and evaluation. Mob Genet Elements 2:171–178. doi:10.4161/mge.21647. 23094235 PMC3469428

[24] Lutkenhaus J, Pichoff S, Du S 2012 Bacterial cytokinesis: from Z ring to divisome. Cytoskeleton (Hoboken) 69:778–790. doi:10.1002/cm.21054. 22888013 PMC3931253

[25] Weart RB, Nakano S, Lane BE, Zuber P, Levin PA 2005 The ClpX chaperone modulates assembly of the tubulin-like protein FtsZ. Mol Microbiol 57:238–249. doi:10.1111/j.1365-2958.2005.04673.x. 15948963 PMC5432201

[26] Dziedzic R, Kiran M, Plocinski P, Ziolkiewicz M, Brzostek A, Moomey M, Vadrevu IS, Dziadek J, Madiraju M, Rajagopalan M 2010 Mycobacterium tuberculosis ClpX interacts with FtsZ and interferes with FtsZ assembly. PLoS One 5:e11058. doi:10.1371/journal.pone.0011058. 20625433 PMC2897852

[27] Sugimoto S, Yamanaka K, Nishikori S, Miyagi A, Ando T, Ogura T 2010 AAA+ chaperone ClpX regulates dynamics of prokaryotic cytoskeletal protein FtsZ. J Biol Chem 285:6648–6657. doi:10.1074/jbc.M109.080739. 20022957 PMC2825460

[28] Santa Maria JP, Sadaka A, Moussa SH, Brown S, Zhang YJ, Rubin EJ, Gilmore MS, Walker S 2014 Compound-gene interaction mapping reveals distinct roles for *Staphylococcus aureus* teichoic acids. Proc Natl Acad Sci U S A 111:12510–12515. doi:10.1073/pnas.1404099111. 25104751 PMC4151746

[29] R Core Team 2014 R: a language and environment for statistical computing. R Foundation for Statistical Computing, Vienna, Austria.

[30] Zwietering MH, Jongenburger I, Rombouts FM, van ’t Riet K 1990 Modeling of the bacterial growth curve. Appl Environ Microbiol 56:1875–1881. 16348228 10.1128/aem.56.6.1875-1881.1990 PMC184525

[31] Giraud C, Hausmann S, Lemeille S, Prados J, Redder P, Linder P 2015 The C-terminal region of the RNA helicase CshA is required for the interaction with the degradosome and turnover of bulk RNA in the opportunistic pathogen *Staphylococcus aureus*. RNA Biol 12:658–674. doi:10.1080/15476286.2015.1035505. 25997461 PMC4615653

[32] Langmead B, Salzberg SL 2012 Fast gapped-read alignment with bowtie 2. Nat Methods 9:357–359. doi:10.1038/nmeth.1923. 22388286 PMC3322381

[33] Li H, Handsaker B, Wysoker A, Fennell T, Ruan J, Homer N, Marth G, Abecasis G, Durbin R; 1000 Genome Project Data Processing Subgroup. 2009 The sequence alignment of map format and SAMtools. Bioinformatics 25:2078–2079. 19505943 10.1093/bioinformatics/btp352 PMC2723002

[34] Quinlan AR, Hall IM 2010 BEDTools: a flexible suite of utilities for comparing genomic features. Bioinformatics 26:841–842. doi:10.1093/bioinformatics/btq033. 20110278 PMC2832824

[35] Aziz RK, Bartels D, Best AA, DeJongh M, Disz T, Edwards RA, Formsma K, Gerdes S, Glass EM, Kubal M, Meyer F, Olsen GJ, Olson R, Osterman AL, Overbeek RA, McNeil LK, Paarmann D, Paczian T, Parrello B, Pusch GD, Reich C, Stevens R, Vassieva O, Vonstein V, Wilke A, Zagnitko O 2008 The RAST server: rapid annotations using subsystems technology. BMC Genomics 9:75. doi:10.1186/1471-2164-9-75. 18261238 PMC2265698

