# Supplementary figures and images for: The Cell Wall Polymer Lipoteichoic Acid Becomes Nonessential in Staphylococcus aureus Cells Lacking the ClpX Chaperone
Source: mBio. 2016 Aug 9;7(4):e01228-16. doi: 10.1128/mBio.01228-16 (PMC4981727; doi:10.1128/mBio.01228-16)

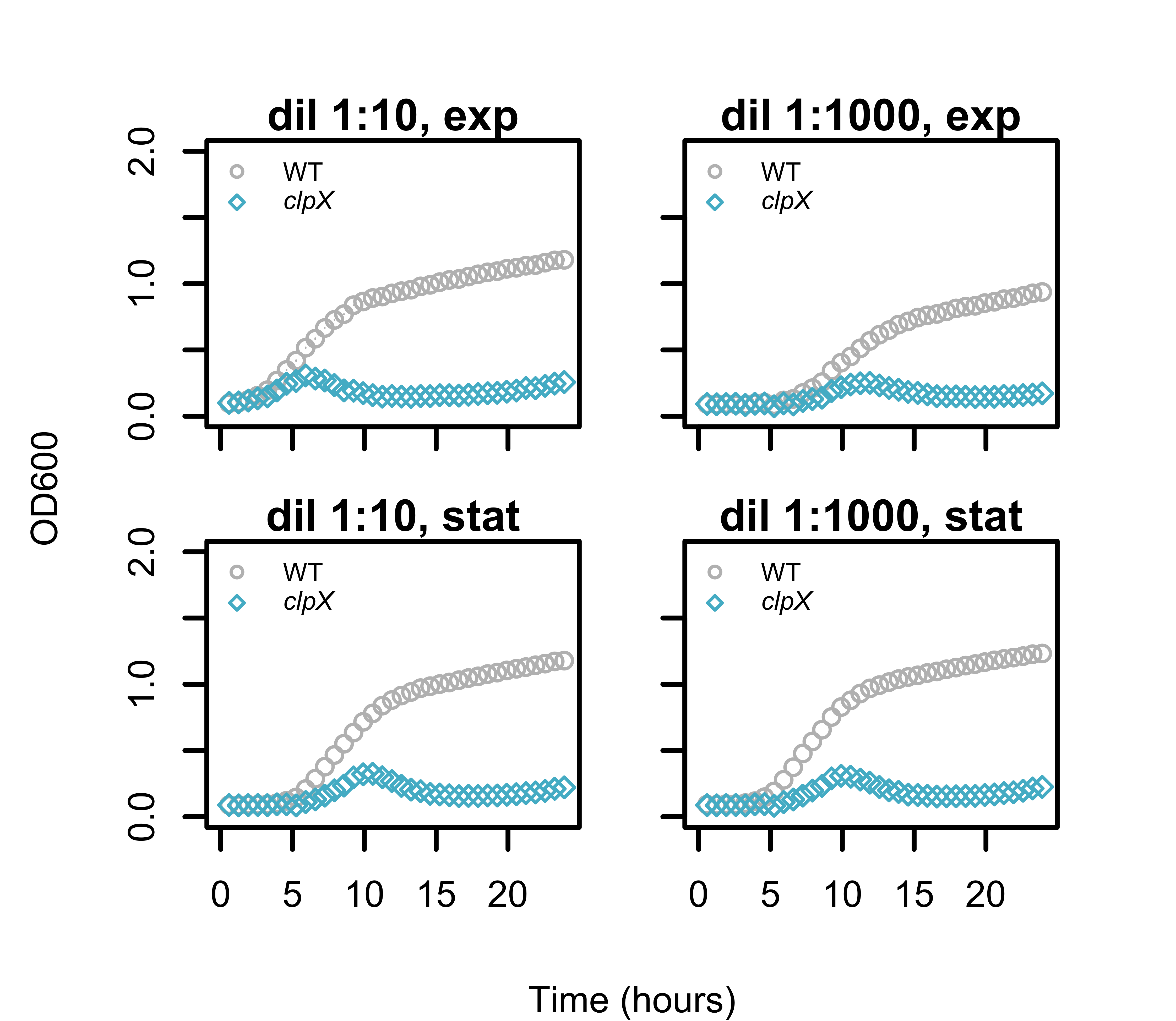

Supplement: Figure S1 — Effect of size and growth phase of inoculum on growth of clpX mutant. S. aureus strains 8325-4 and 8325-4 clpX were grown to exponential (OD, ~0.1; “exp”) or stationary (overnight culture; “stat”) phase at 37°C and diluted (1:10 or 1:1,000) as indicated into 300 µl TSB, and growth was measured at 30°C in a Bioscreen C instrument. Values represent mean OD readings ± standard deviations (error bars are smaller than the symbols) of the results from three biological replicates. Download [file mbo004162933sf1.tif]

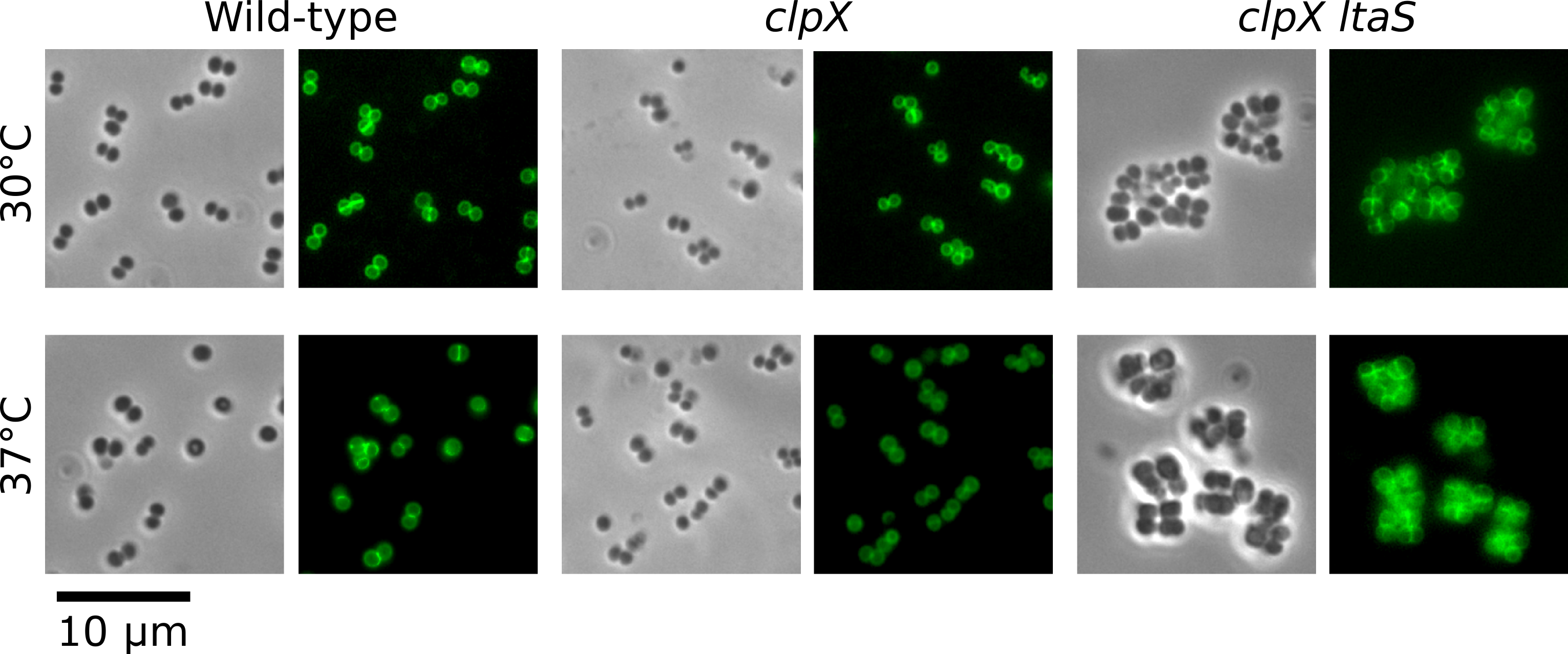

Supplement: Figure S2 — Microscopic analysis of the wild-type, clpX-inactivated, and LTA-negative strains. The S. aureus SA564 wild type, the SA564 clpX strain, and the 564-25-2 (SA564 clpX ltaSH476Q) strain were grown in TSB to the log phase at either 30°C or 37°C, samples were prepared for microscopy analysis, and bacteria were stained with vancomycin-BODIPY. Left panels show phase-contrast images; right panels show fluorescence images. Download [file mbo004162933sf2.tif]

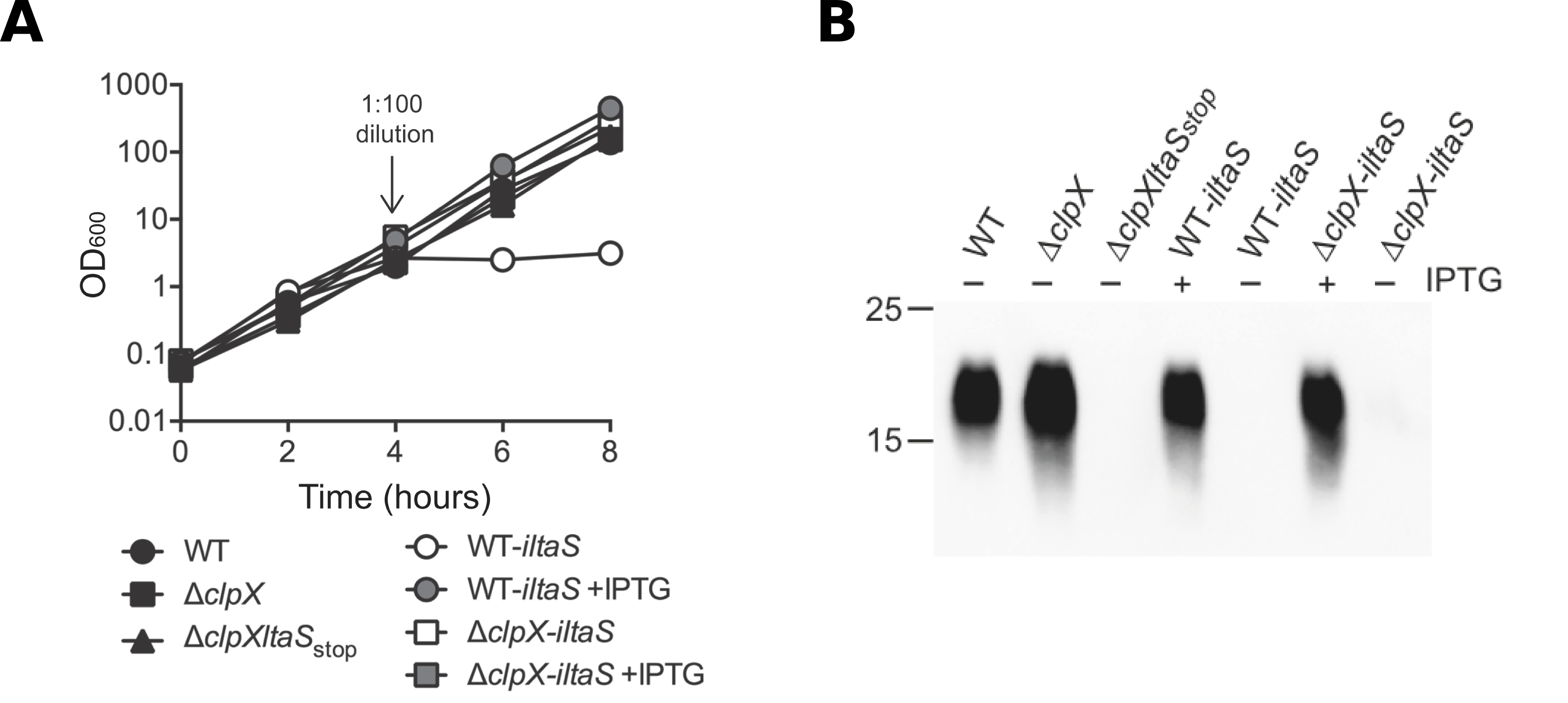

Supplement: Figure S3 — (A) Growth curves of S. aureus 8325-4 and derived strains. Overnight cultures of S. aureus strains 8325-4 (WT), 8325-4-ΔclpX, 8325-4-ΔclpX ltaSstop, 8325-4-iltaS (WT-iltaS), and 8325-4ΔclpX-iltaS were harvested, washed, and diluted 1:1,000 into TSB substituted with 1 mM IPTG. Cultures were incubated at 37°C, and OD600 values were recorded. To maintain the cultures in log phase, the cultures were back diluted 1:100 at the 4-h time point into 5 ml fresh TSB (supplemented when required with 1 mM IPTG) and growth continued for an additional 4 h. The average OD600 values and standard deviations from three independent experiments are plotted. (B) Analysis of LTA synthesis. Extracts obtained from S. aureus strains 8325-4 (WT), 8325-4-ΔclpX, 8325-4-ΔclpX ltaSstop, 8325-4-iltaS (WT-iltaS), and 8325-4ΔclpX-iltaS were separated by SDS/PAGE, electrotransferred to a PVDF membrane, and subjected to immunoblotting using an LTA-specific antibody. Download [file mbo004162933sf3.tif]

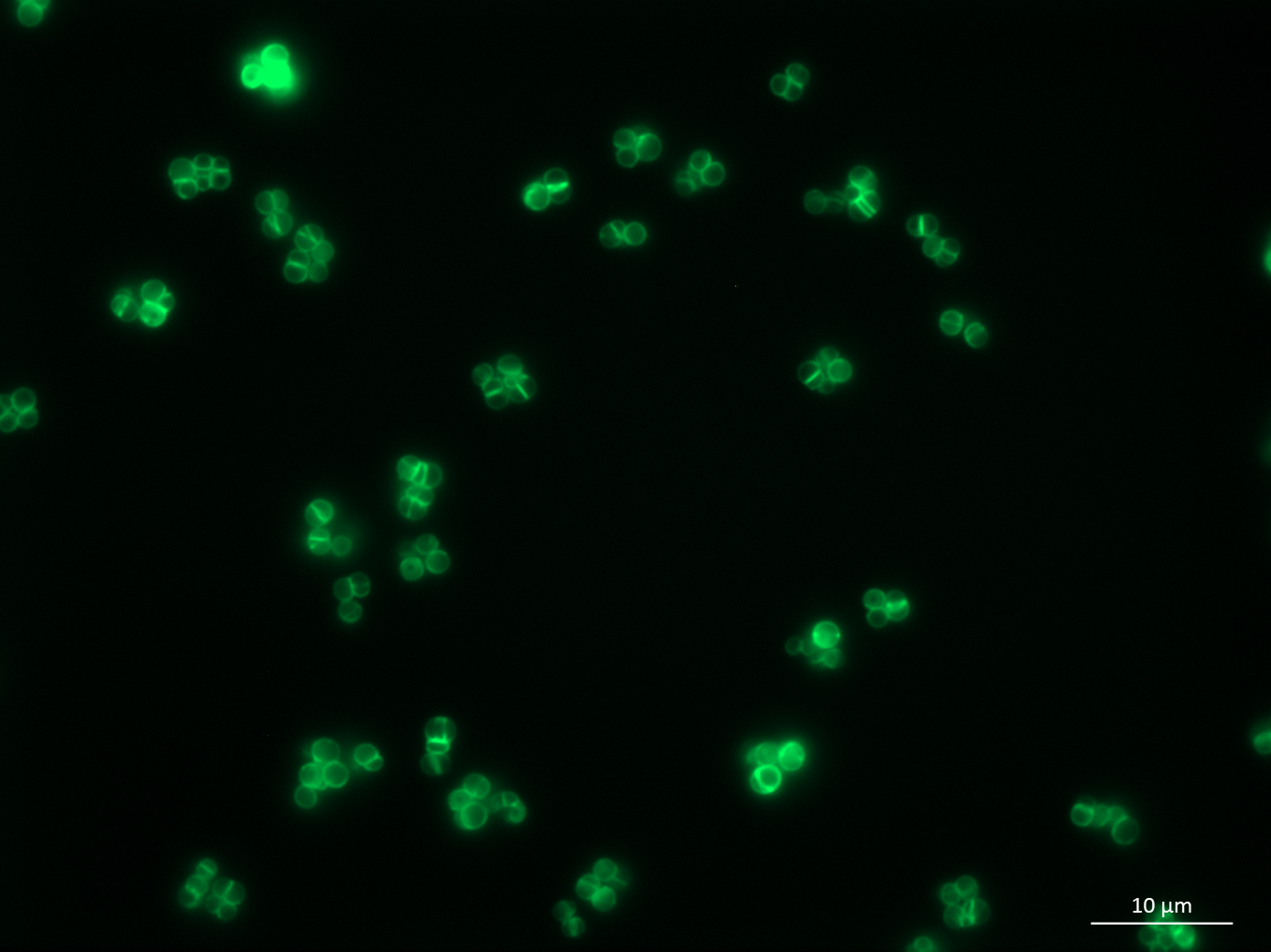

Supplement: Figure S4 — Fluorescence microscopic analysis of strain 8325-4-iltaS grown to exponential phase at 37°C in the presence of 1 mM IPTG, and stained with BODIPY-vancomycin. Bacteria were grown and prepared for fluorescence microscopy as described in Materials and Methods. Download [file mbo004162933sf4.tif]

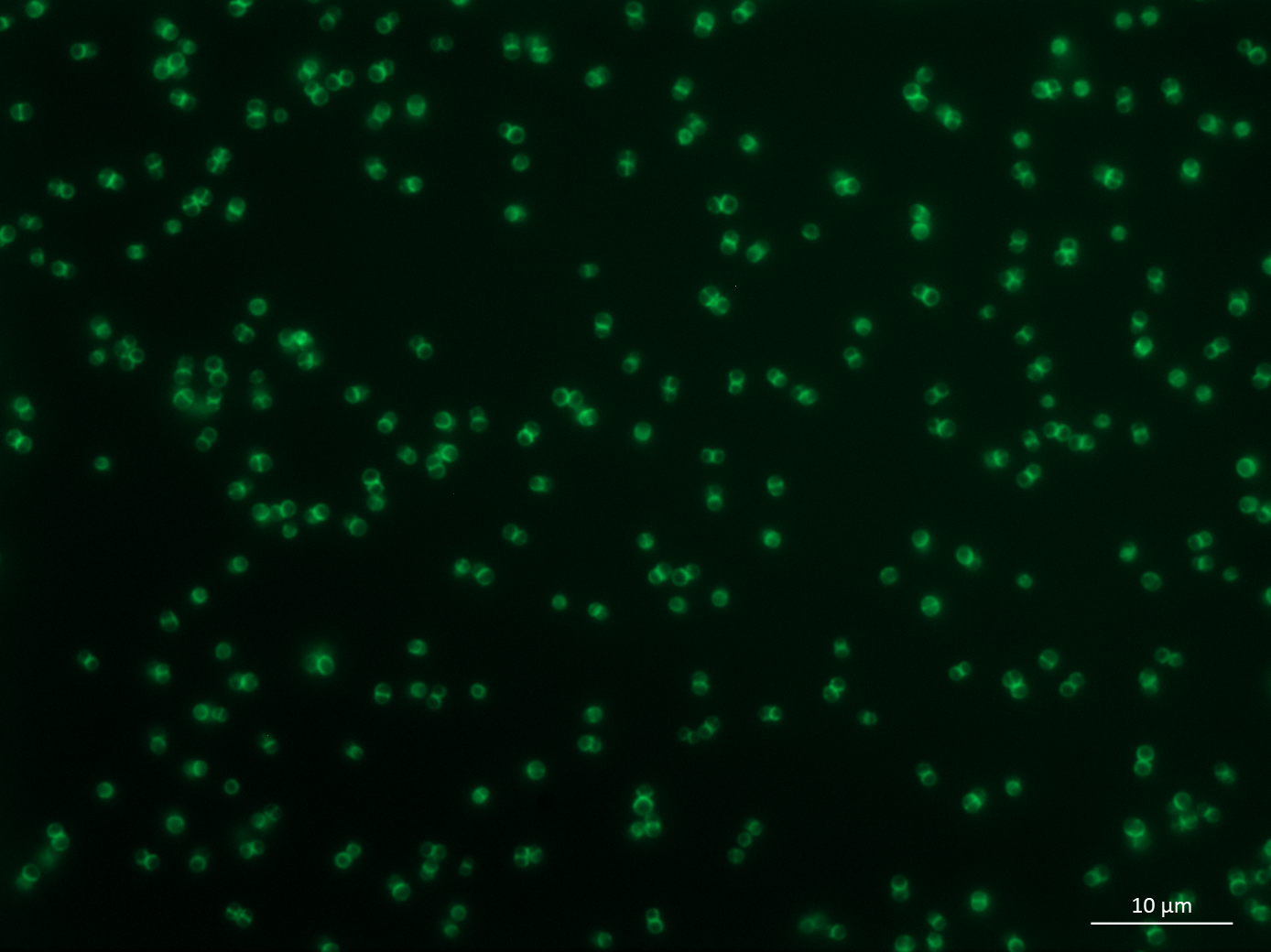

Supplement: Figure S5 — Fluorescence microscopic analysis of strain 8325-4-clpX-iltaS grown to exponential phase at 37°C in the presence of 1 mM IPTG and stained with BODIPY-vancomycin. Bacteria were grown and prepared for fluorescence microscopy as described in Materials and Methods. Download [file mbo004162933sf5.tif]

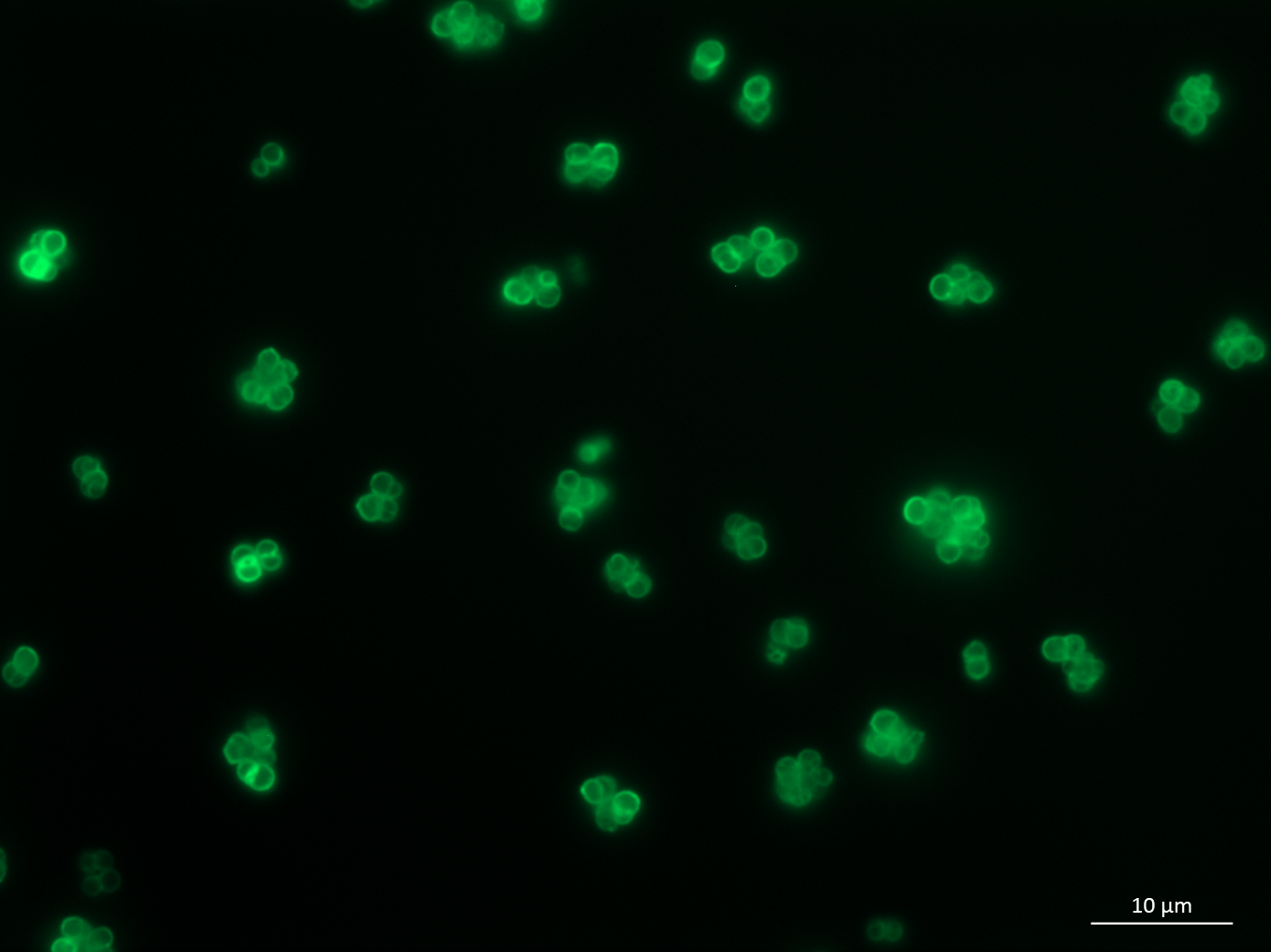

Supplement: Figure S6 — Fluorescence microscopic analysis of strain 8325-4-iltaS grown to exponential phase at 37°C in the absence of IPTG and stained with BODIPY-vancomycin. Bacteria were grown and prepared for fluorescence microscopy as described in Materials and Methods. Download [file mbo004162933sf6.tif]

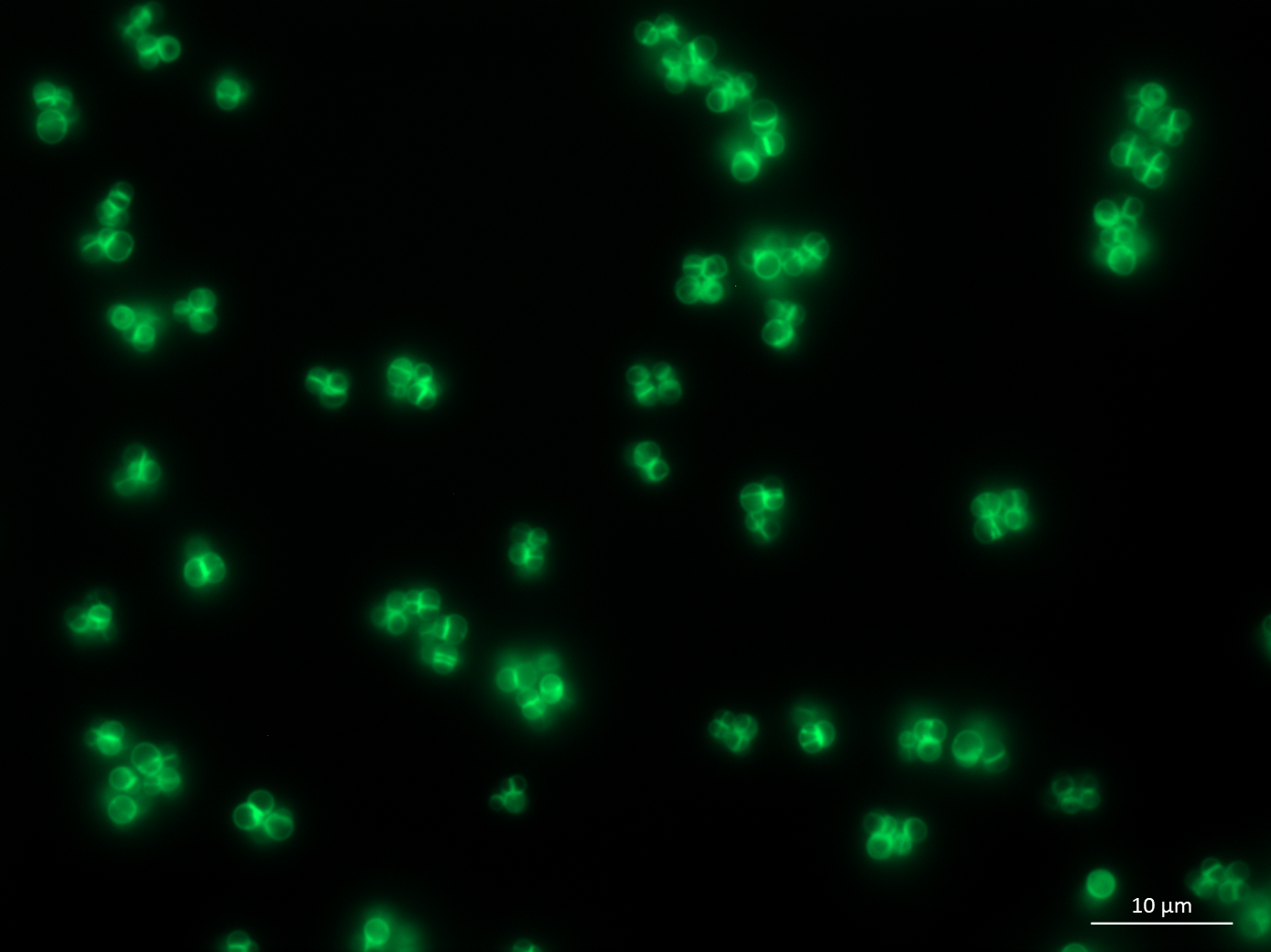

Supplement: Figure S7 — Fluorescence microscopic analysis of strain 8325-4-clpX-iltaS grown to exponential phase at 37°C in the absence of IPTG and stained with BODIPY-vancomycin. Bacteria were grown and prepared for fluorescence microscopy as described in Materials and Methods. Download [file mbo004162933sf7.tif]
