# Supplementary material for: The Cell Wall Polymer Lipoteichoic Acid Becomes Nonessential in Staphylococcus aureus Cells Lacking the ClpX Chaperone
Source: mBio. 2016 Aug 9;7(4):e01228-16. doi: 10.1128/mBio.01228-16 (PMC4981727; doi:10.1128/mBio.01228-16)
Supplement: Table S1 — Bacterial strains, plasmids, and primers used in this study. [file mbo004162933st1.pdf]

Table S1: Bacterial strains, plasmids and primers used in this study

Strain	Description	Source
***E. coli* strains**		
XL1 Blue	Cloning strain	Stratagene
***S. aureus* strains**		
RN4220	Restriction deficient derivative of 8325-4	(1)
KB1475	RN4220 pKB1469	This study
KB1478	RN4220 pRAB12-lacZ	This study
SA564	Low-passage clinical isolate	(2)
HI2781	SA564Δ*clpX*	(3)
HI2726	SA564Δ*clpP*	(4)
KB1090	564-25-2: SA564Δ*clpX* $ltaS_{H476Q}$ suppressor mutant	This study
KB1501	SA564Δ*clpX ltaS::erm*	This study
KB1492	SA564 pRAB11	This study
KB1486	SA564 pKB1469	This study
KB1496	SA564Δ*clpX* pRAB11	This study
KB1490	SA564Δ*clpX* pKB1469	This study
Newman	Clinial isolate (ATCC 25904)	(5)
HI3461	NewmanΔ*clpX*	This study
HI2564	NewmanΔ*clpP*	(4)
8325-4	MSSA strain cured of prophages	(6)
HI2209	8325-4Δ*clpX*	(7)
HI2300	8325-4Δ*clpP*	(7)
KB1133	83-25C: 8325-4Δ*clpX* $ltaS_{382STOP}$ suppressor mutant	This study
KB1262	8325-4Δ*clpX* $ltaS_{122STOP}$ suppressor mutant	This study
KB1241	8325-4Δ*clpX ermB*	This study
KB1506	8325-4Δ*clpX clpP*::ΦΝΣ	This study
KB1510	8325-4 $ltaS_{382STOP}$ Δ*clpX* pRAB12-lacZ	This study

KB1509	8325-4 *ltaS*$_{382STOP}$ Δ*clpX* pKB1469	This study
ANG4052	8325-4 *iltaS*	This study
ANG4053	8325-4 Δ*clpX-iltaS*	This study
JE2	CA-MRSA strain USA300 LAC cured of plasmids	(8)
HI3393	JE2Δ*clpX*	(9)
NE912	JE2*clpP*::ΦΝΣ	Nebraska Transposon Mutant Library (http://www.narsa.net)
KB1399	JE2*clpP*::ΦΝΣ, transduced from NE912	(9)
LAC*	Erm sensitive CA-MRSA LAC strain (AH1263)	(10)
ANG1961	LAC* Δ*gdpP::kan*	(11)
ANG2142	US3: LAC* *ltaS::erm* suppressor	(11)
**Plasmids**		
pRAB11	Expression vector with anhydrotetracycline inducable *xyl/tet* promoter. Cam^R	(12)
pRAB12-lacZ	Expression vector with anhydrotetracycline inducable *xyl/tet* promoter. Cam^R	(12)
pKB1469	pRAB12-lacZ containing *clpX* under control of the *xyl/tet* promoter	This study
**Primers**		
Name	Sequence (5' - 3')	
KB71F	TATCTAATTTATTCAAGTACTTTCGC	
KB71R	AACTTGTTAAGGCTTTGATGAC	
KB73F	CAGAATTATTAACACGTACATTTGAC	
KB76F	GTTTCATTTCCATTTGCTATACCTCC	
KB76R	TATTCATCGCACGTATTACTTCCA	
KB110F	GGACTGGCCGCCTAATAATAAAA	
KB110R	TTTGCGGGAGTAGTTCAACTTTT	
KB111F	CCGAATTCTTATAACACATCAATGATTAAGCTG	
KB111R	CCAGATCTAACGAATAGGGGTGTAAAAAGAATG	

KB121F	GACGATGCAGAACAACGTG
KB121R	CCATACCCTGGAACATCCAC
KB122F	CTTCTGGGCGAATTTGTGCT
KB122R	ACAAGGTGGACGCAAACATC
saclpX385f	GACGATGCAGAACAACGTG
saclpX2447r	CCATACCCTGGAACATCCAC
saclpP413f	AGAATTATAAGTAAGAAG
saclpP1644r	ATTGACACCTTGGTTTACTC

1. **Kreiswirth BN**, **Löfdahl S**, **Betley MJ**, **O'Reilly M**, **Schlievert PM**, **Bergdoll MS**, **Novick RP**. 1983. The toxic shock syndrome exotoxin structural gene is not detectably transmitted by a prophage. Nature **305**:709–712.

2. **Somerville GA**, **Beres SB**, **Fitzgerald JR**, **DeLeo FR**, **Cole RL**, **Hoff JS**, **Musser JM**. 2002. In vitro serial passage of Staphylococcus aureus: changes in physiology, virulence factor production, and agr nucleotide sequence. J Bacteriol **184**:1430–1437.

3. **Jelsbak L**, **Ingmer H**, **Valihrach L**, **Cohn MT**, **Christiansen MHG**, **Kallipolitis BH**, **Frees D**. 2010. The chaperone ClpX stimulates expression of Staphylococcus aureus protein A by Rot dependent and independent pathways. PLoS ONE **5**:e12752.

4. **Frees D**, **Andersen JH**, **Hemmingsen L**, **Koskenniemi K**, **Bæk KT**, **Muhammed MK**, **Gudeta DD**, **Nyman TA**, **Sukura A**, **Varmanen P**, **Savijoki K**. 2012. New insights into Staphylococcus aureus stress tolerance and virulence regulation from an analysis of the role of the ClpP protease in the strains Newman, COL, and SA564. J Proteome Res **11**:95–108.

5. **Duthie ES**, **Lorenz LL**. 1952. Staphylococcal coagulase; mode of action and antigenicity. J Gen Microbiol **6**:95–107.

6. **Bæk KT**, **Frees D**, **Renzoni A**, **Barras C**, **Rodriguez N**, **Manzano C**, **Kelley WL**. 2013. Genetic variation in the Staphylococcus aureus 8325 strain lineage revealed by whole-genome sequencing. PLoS ONE **8**:e77122.

7. **Frees D**, **Qazi S**, **Hill PJ**, **Ingmer H**. 2003. Alternative roles of ClpX and ClpP in Staphylococcus aureus stress tolerance and virulence. Mol Microbiol **48**:1565–1578.

8. **Fey PD**, **Endres JL**, **Yajjala VK**, **Widhelm TJ**, **Boissy RJ**, **Bose JL**, **Bayles KW**. 2013. A genetic resource for rapid and comprehensive phenotype screening of nonessential Staphylococcus aureus genes. MBio **4**:e00537–12.

9. **Bæk KT**, **Gründling A**, **Mogensen RG**, **Thøgersen L**, **Petersen A**, **Paulander W**, **Frees D**. 2014. β-Lactam resistance in methicillin-resistant Staphylococcus aureus USA300 is increased by inactivation of the ClpXP protease. Antimicrob Agents Chemother **58**:4593–4603.

10. **Boles BR**, **Thoendel M**, **Roth AJ**, **Horswill AR**. 2010. Identification of genes involved in polysaccharide-independent Staphylococcus aureus biofilm formation. PLoS ONE **5**:e10146.

11. **Corrigan RM**, **Abbott JC**, **Burhenne H**, **Kaever V**, **Gründling A**. 2011. c-di-AMP is a new second messenger in Staphylococcus aureus with a role in controlling cell size and envelope stress. PLoS Pathog **7**:e1002217.

12. **Helle L**, **Kull M**, **Mayer S**, **Marincola G**, **Zelder M-E**, **Goerke C**, **Wolz C**, **Bertram R**. 2011. Vectors for improved Tet repressor-dependent gradual gene induction or silencing in Staphylococcus aureus. Microbiology (Reading, Engl) **157**:3314–3323.
