# Supplementary material for: The Cell Wall Polymer Lipoteichoic Acid Becomes Nonessential in Staphylococcus aureus Cells Lacking the ClpX Chaperone
Source: mBio. 2016 Aug 9;7(4):e01228-16. doi: 10.1128/mBio.01228-16 (PMC4981727; doi:10.1128/mBio.01228-16)
Supplement: Text S1 — Supplementary Materials and Methods. Download [file mbo004162933s1.pdf]

Text S1: Supplementary Materials and Methods

Construction of strains and plasmids

Primers used in this study are listed in Table S1. Plasmid pKB1469 was created by amplifying the *clpX* gene with primers KB111F/KB111R using SA564 chromosomal DNA as template. The PCR product was digested with *Bgl*II and *Eco*RI and cloned into pRAB12-lacZ (1) digested with the same enzymes, and the insert was sequence-verified with primers KB120F/KB120R.

The *ltaS* gene was disrupted in the SA564 *clpX* strain by transduction with bacteriophage Φ85 using strain ANG2142 as donor strain, selecting for erythromycin (10 μg/ml) resistance. The insertion in *ltaS* in the resulting strain KB1501 was verified by PCR with primers KB71F/KB71R, and the *clpX* deletion was verified with primers KB111F/KB111R.

The *clpP* gene was disrupted in the 8325-4 *clpX* strain by transduction with bacteriophage Φ11 using NE912 (JE2*clpP*::ΦNΣ) as donor strain, selecting for erythromycin (5 μg/ml) resistance. The insertion in *clpP* in the resulting strain KB1506 was verified by PCR with primers saclpP413f/saclpP1644r, and the *clpX* deletion was verified with primers KB121F/KB121R. Plasmid pKB1469 and the vector pRAB12-lacZ were introduced into strain 83-25-C by transduction with bacteriophage Φ85 using KB1475 and KB1478 as donor strains, selecting for chloramphenicol (10 μg/ml) resistance. The chromosomal *clpX* deletion in the resulting strains KB1509 and KB1510 was verified by PCR with primers KB121F/KB121R, and the presence of the

complementing *clpX* gene was tested with primers KB122F/KB122R binding internally in the region which is deleted in the chromosomal *clpX* gene.

S. aureus strain KB1241 was constructed by inserting an *ermB* marker into strain 8325-4 eight kb from *clpX* between the convergently transcribed genes with locus tags SAOUHSC_1768 and SAOUHSC_1769. KB1241 was constructed as detailed in (2) for KB1239, but with *ermB* transcribed in the opposite direction.

The *clpX* gene was deleted in *S. aureus* strain Newman by transduction with bacteriophage Φ11, using an *ermB*-tagged *S. aureus* 8325-4Δ*clpX* mutant (KB1257) as the donor strain for the transduction, as described previously (2).

Strain 8325-4-*iltaS* (ANG4052) and 8325-4-*clpX*-*iltaS* (ANG4053) were constructed by moving the erythromycin marked IPTG inducible *ltaS* gene from strain RN4220-*iltaS* (3) by transduction with bacteriophage Φ85 into strains 8325-4 and 8325-4-*clpX*, respectively.

Western blotting

Western immunoblotting of LTA was performed as described previously (3). In brief, LTA was extracted from 1 ml cultures and the samples were normalized based on optical density (OD_{600}) readings of the original cultures. Samples were boiled for 20 min, centrifuged at 17,000 x g for 5 min and 10 µl aliquots separated on 15% SDS-PAGE gels and subsequently transferred to a PVDF membrane. LTA was detected using the monoclonal polyglycerolphosphate-specific LTA antibody (1:4,000; Clone 55 from Hycult Biotechnology) and the HRP-conjugated anti-mouse IgG (1:10,000; Cell Signaling Technologies, USA) and blots were developed by enhanced

chemiluminescence. A representative blot from three independent experiments is shown.

For LtaS detection, cells from 1 ml of culture were harvested by centrifugation at 5,000 x g, cellular extracts were prepared by lysostaphin treatment, and volumes normalized to the optical density of the initial culture were loaded onto 4 to 12% SDS-PAGE gels (NuPAGE). Western blotting was performed as previously described (4) using an antibody against LtaS (5). A representative blot from three independent experiments is shown.

Autolytic activity

Analysis of Triton X-100 induced autolysis was performed as described previously (2). In short, overnight cultures were diluted into fresh TSB medium and grown to an OD_{600} of 1. Cells were harvested at 8000 x g, washed twice in 10 mM sodium phosphate buffer pH 7.0, and twice in ice-cold ddH2O before suspending in 10 mM sodium phosphate buffer, pH 7.0 containing 0.05% (v/v) Triton X-100. Suspensions were incubated at 37°C and autolysis monitored by measuring OD_{600} values at the indicated time points.

Zymographic analyses were performed as described previously (2). In short, cultures were grown and harvested as above, and the pellet was suspended in SDS sample buffer and boiled for 20 min. Boiled samples were centrifuged at 17000 x g, and volumes of supernatant normalized to the optical density of the initial culture were loaded onto 7.5% SDS-PAGE gels containing 3% (w/v) heat-killed *S. aureus* SA564 cells. Gels were washed twice in ddH2O before overnight incubation in 0.2 M phosphate

buffer pH 7.0 at 37°C. Gels were subsequently stained with 0.1% methylene blue. Densitometric analysis was performed in ImageJ.

Antibiotic susceptibility testing

MICs were determined by Etest (bioMérieux) according to the manufacturer's recommendations using *S. aureus* strain ATCC 29213 as reference.

c-di-AMP measurements

Cultures of *S. aureus* cells were grown in 5 ml TSB for 4-5 h, back-diluted to an OD_{600} of 0.05 in 50 ml TSB, and grown for 16 h at 37°C with aeration. Cultures were adjusted to an approximate OD_{600} of 2 and bacteria from a 10 ml culture aliquot were also collected by centrifugation, washed and freeze dried to determine the dry weight for normalization purposes. Preparation of bacterial extracts, labelling of c-di-AMP, and quantification of c-di-AMP by LC-MS/MS was carried out as described previously (6).

Trypsin shaving, LC-MS/MS and proteome bioinformatics

The wild type (SA564) and the mutant strains (*clpX* and *clpX ltaS*) were grown in duplicates (OD_{600} ~ 0.05, t = 0) in TSB at 30°C or 37°C under vigorous shaking (250 rpm) to exponential phase at 37 °C (OD600~ 0.8) and at 30 °C (OD600~0.5 for wild type and 0.3 for the mutant strains). Samples (20 mL for cells at 37 °C and 40 mL for cells at 30 °C) were removed and cells were harvested by centrifugation (4000 x g, 5 min, 4°C). Pelleted cells were washed two times in 1 mL TEAB (50 mM triethyl ammonium bicarbonate buffer, pH 8.0) and then gently suspended in 100 µl of TEAB. The cells in TEAB were mixed with 100 ng of sequencing grade modified porcine trypsin

(Promega) and the digestions were incubated at 37°C for 15 min. Released peptides and trypsin were recovered by filtration through a 0.2-°m pore size acetate membranes by centrifugation (7,000 x g, 2 min, +4°C) and the digestions were further incubated for 16 h at 37°C. Digestions were stopped by addition of trifluoracetic acid to a final concentration of 0.6%. The peptide concentrations were measured using the Nano-Drop (ND 1000, Fisher Scientific) at 280 nm.

Trypsin-digested peptides were purified using ZipTips (C18) (Millipore). Equal amount of the peptide samples were subjected to LC-MS/MS identification using an Ultimate 3000 nano-LC (Dionex) and QSTAR Elite hybrid quadrupole TOF mass spectrometer (Applied Biosystems / MDS Sciex) with nano-ESI ionization as previously described (7). The MS/MS data were searched against the in-house SA564 database (2577 entries) build from a draft genome sequence (Frees et al., unpublished) using the Mascot (version 2.4.0) and Paragon search engines (version 4.0.0.0) through the ProteinPilotTM (version 4.0) software. The parameters for Paragon searches included the Rapid search mode and the carbamidomethyl modification of cysteine as a fixed and oxidation of methionine as a variable modification. The Compid tool (8) was used to parse and compile significant hits from both search data. Protein identifications with Mascot identification scores ≥ 40 and $p < 0.05$ and/or Paragon Unused ProtScores ≥ 1.3 and $p < 0.05$ were considered reliable.

Theoretical molecular weights (MW) and isoelectric points (pI) for the identified proteins were acquired with ProMoST Protein Modification Screening tool (9). The cellular location for each protein was predicted using the PSORTb. The SignalP 4.0 Server was used to detect potential signal sequences in each protein sequence, whereas proteins

exploiting non-classical secretion mechanisms were predicted using the SecretomeP 2.0 Server (10, 11). The presence of potential transmembrane domains was determined using the TMHMM Server v. 2.0 (12).

1. **Helle L**, **Kull M**, **Mayer S**, **Marincola G**, **Zelder M-E**, **Goerke C**, **Wolz C**, **Bertram R**. 2011. Vectors for improved Tet repressor-dependent gradual gene induction or silencing in Staphylococcus aureus. Microbiology (Reading, Engl) **157**:3314–3323.

2. **Bæk KT**, **Gründling A**, **Mogensen RG**, **Thøgersen L**, **Petersen A**, **Paulander W**, **Frees D**. 2014. β-Lactam resistance in methicillin-resistant Staphylococcus aureus USA300 is increased by inactivation of the ClpXP protease. Antimicrob Agents Chemother **58**:4593–4603.

3. **Gründling A**, **Schneewind O**. 2007. Synthesis of glycerol phosphate lipoteichoic acid in Staphylococcus aureus. Proc Natl Acad Sci USA **104**:8478–8483.

4. **Jelsbak L**, **Ingmer H**, **Valihrach L**, **Cohn MT**, **Christiansen MHG**, **Kallipolitis BH**, **Frees D**. 2010. The chaperone ClpX stimulates expression of Staphylococcus aureus protein A by Rot dependent and independent pathways. PLoS ONE **5**:e12752.

5. **Lu D**, **Wörmann ME**, **Zhang X**, **Schneewind O**, **Gründling A**, **Freemont PS**. 2009. Structure-based mechanism of lipoteichoic acid synthesis by Staphylococcus aureus LtaS. Proc Natl Acad Sci USA **106**:1584–1589.

6. **Corrigan RM**, **Abbott JC**, **Burhenne H**, **Kaever V**, **Gründling A**. 2011. c-di-AMP is a new second messenger in Staphylococcus aureus with a role in controlling cell size and envelope stress. PLoS Pathog **7**:e1002217.

7. **Savijoki K**, **Lietzén N**, **Kankainen M**, **Alatossava T**, **Koskenniemi K**, **Varmanen P**, **Nyman TA**. 2011. Comparative proteome cataloging of Lactobacillus rhamnosus strains GG and Lc705. J Proteome Res **10**:3460–3473.

8. **Lietzén N**, **Natri L**, **Nevalainen OS**, **Salmi J**, **Nyman TA**. 2010. Compid: a new software tool to integrate and compare MS/MS based protein identification results from Mascot and Paragon. J Proteome Res **9**:6795–6800.

9. **Halligan BD**, **Ruotti V**, **Jin W**, **Laffoon S**, **Twigger SN**, **Dratz EA**. 2004. ProMoST (Protein Modification Screening Tool): a web-based tool for mapping protein modifications on two-dimensional gels. Nucleic Acids Res **32**:W638–44.

10. **Petersen TN**, **Brunak S**, **Heijne G von**, **Nielsen H**. 2011. SignalP 4.0: discriminating signal peptides from transmembrane regions. Nat Methods **8**:785–786.

11. **Bendtsen JD**, **Kiemer L**, **Fausbøll A**, **Brunak S**. 2005. Non-classical protein secretion in bacteria. BMC Microbiol **5**:58.

12. **Krogh A**, **Larsson B**, **Heijne G von**, **Sonnhammer EL**. 2001. Predicting transmembrane protein topology with a hidden Markov model: application to complete genomes. J Mol Biol **305**:567–580.
